# MtDNA control region variation affirms diversity and deep sub-structure in populations from southern Africa

**DOI:** 10.1186/1471-2148-13-56

**Published:** 2013-02-27

**Authors:** Carina M Schlebusch, Marlize Lombard, Himla Soodyall

**Affiliations:** 1 Human Genomic Diversity and Disease Research Unit, Division of Human Genetics, School of Pathology, Faculty of Health Sciences, University of the Witwatersrand and the National Health Laboratory Service, Johannesburg, 2000, South Africa; 2 Department of Evolutionary Biology, Evolutionary Biology Centre, Uppsala University, Norbyvägen 18D, Uppsala, SE, 752 36, Sweden; 3 Department of Anthropology and Development Studies, University of Johannesburg, Auckland Park Campus, PO Box 524, Johannesburg, 2006, South Africa

**Keywords:** mtDNA, L0d, L0k, Khoisan-speakers, Khoe-San, San, Southern Africa, African history, Mitochondrial haplogroup

## Abstract

**Background:**

The current San and Khoe populations are remnant groups of a much larger and widely dispersed population of hunter-gatherers and pastoralists, who had exclusive occupation of southern Africa before the influx of Bantu-speakers from 2 ka (ka = kilo annum [thousand years] old/ago) and sea-borne immigrants within the last 350 years. Here we use mitochondrial DNA (mtDNA) to examine the population structure of various San and Khoe groups, including seven different Khoe-San groups (Ju/’hoansi, !Xun, /Gui+//Gana, Khwe, ≠Khomani, Nama and Karretjie People), three different Coloured groups and seven other comparative groups. MtDNA hyper variable segments I and II (HVS I and HVS II) together with selected mtDNA coding region SNPs were used to assign 538 individuals to 18 haplogroups encompassing 245 unique haplotypes. Data were further analyzed to assess haplogroup histories and the genetic affinities of the various San, Khoe and Coloured populations. Where possible, we tentatively contextualize the genetic trends through time against key trends known from the archaeological record.

**Results:**

The most striking observation from this study was the high frequencies of the oldest mtDNA haplogroups (L0d and L0k) that can be traced back in time to ~100 ka, found at high frequencies in Khoe-San and sampled Coloured groups. Furthermore, the L0d/k sub-haplogroups were differentially distributed in the different Khoe-San and Coloured groups and had different signals of expansion, which suggested different associated demographic histories. When populations were compared to each other, San groups from the northern parts of southern Africa (Ju speaking: !Xun, Ju/’hoansi and Khoe-speaking: /Gui+//Gana) grouped together and southern groups (historically Tuu speaking: ≠Khomani and Karretjie People and some Coloured groups) grouped together. The Khoe group (Nama) clustered with the southern Khoe-San and Coloured groups. The Khwe mtDNA profile was very different from other Khoe-San groups with high proportions of Bantu-speaking admixture but also unique distributions of other mtDNA lineages.

**Conclusions:**

On the whole, the research reported here presented new insights into the multifaceted demographic history that shaped the existing genetic landscape of the Khoe-San and Coloured populations of southern Africa.

## Background

The term “Khoe-San” represents groups with two distinct lifeways, the Khoi (old Nama word) or Khoe (modern Nama word), who were traditionally the pastoralist groups and the San, which included the hunter-gatherer groups [1,2]. This grouping was made according to the conventional division that existed between hunter-gatherers and pastoralists at the time of colonization. Whether the grouping is a true reflection of subdivision remains a topic of debate [3,4]. The current distribution of Khoe-San groups comprises a wide geographic region from southern Angola in the north to the Western Cape Province (South Africa) in the south. They live among, and to some extent are admixed with, the various surrounding Bantu-speaking populations [3,5,6]. Linguistically, Khoe-San groups are divided into northern Khoisan-speaking groups (Ju division) and southern Khoisan-speaking groups (Tuu division) with an additional linguistic group (Khoe-Kwadi) associated with some Khoe-speaking San groups in Botswana and the Khoe herders of South Africa and Namibia (such as the Nama) [7,8].

Mitochondrial DNA (mtDNA) studies showed that Khoe-San populations tend to carry unique and more divergent lineages than the mtDNA lineages associated with Bantu-speakers [9-12]. In fact, the deepest mtDNA clades known among modern humans (L0d and L0k), are found commonly and at their highest frequencies in the Khoe-San. Only few studies thus far focused on the maternal genetic history of the Khoe-San [9-12] (Additional file 1: Table S1). These studies covered only three groups of San, including, the two Ju-speaking groups: the !Xun that were originally from Angola (now located in Platfontein, South Africa [13]) and the linguistically closely related Ju/’hoansi (from northern Botswana and Namibia), and the Khoe-speaking San group, the Khwe (also originally from Angola but now located in Platfontein). All three of these groups were originally from either Angola or northern Namibia, positioning them in the northern parts of the original distribution of the Khoe-San. This left a gap, with no studies conducted on groups representative of the southern San and Khoe, that we aim to start addressing with this contribution. It is, however, becoming increasingly difficult to study the history of the Khoe-San, as groups are losing their cultural identities, socio-economies and languages, and are integrating into surrounding groups.

Although the San and Khoe groups are rather small populations today, their genetic contribution to some of the Coloured populations of South Africa has been substantial [14-16]. To understand the underlying genetic factors in an admixed population such as the Coloured groups of South Africa, it is essential to study the parental populations. By examining the mtDNA variation in several San and Khoe groups from different regions in southern Africa as well as three selected Coloured groups from South Africa, we provide insight into how females have contributed to the genetic affinities of the Khoe-San and some Coloured groups, as well as an opportunity to further examine the evolutionary history of mtDNA haplogroups L0d and L0k.

## Results

The 538 samples used in mtDNA analysis (Table 1, Additional file 2: Figure S1) were first classified into macro-haplogroups using a minisequencing method, and finer scale classification was achieved by analyzing HVS-I and II sequences. A total of 1124 bp in a combined HVS-I and II were analyzed and there were 205 (18.2%) variable positions in the combined sequence (HVS-I had 21.3% and HVS-II had 15.1%). The transition:transversion ratio was 5.6:1. Insertions were observed at four positions (291, 455, 523, 573), whereas deletions occurred at seven positions (16183, 16179, 16325, 247, 249, 498, 523). All deletions involved 1 bp except the 523 region of HVS-II that contained an “AC” repeat motif that were inserted or deleted in several sequences. Insertions involved 1 bp insertions at 291 and 455; one sequence had a 2 bp insertion at 455. All insertions in the poly C repeat track at position 568–573 where taken as a 1 bp C insertion.

**Table 1:** Sample group details

**Group name**	**Group code**	**Main group**	**Language grouping**	**Place of sampling (Country)**	**Place of origin if different from place of sampling**	**N**	**Traditional subsistence**
Karoo Coloured	COL	Coloured	Mixed	Colesberg (SA)		77	Mixed
Cape Coloured	CAC	Coloured	Mixed	Wellington (SA)		20	Mixed
Northern Cape Coloured	CNC	Coloured	Mixed	Askham (SA)		40	Mixed
Karretjie people	KAR	Khoe-San	Descendents of Tuu speakers	Colesberg (SA)		30	Hunter-gatherer and Herder
≠Khomani	KHO	Khoe-San	Descendents of Tuu speakers	Askham (SA)		57	Hunter-gatherer and Herder
//Xegwi *	XEG *	Khoe-San*	Descendents of Tuu speakers	Chrissiesmeer (SA)		3	Hunter-gatherer
Duma San *	DUM *	Khoe-San*	Descendents of Tuu speakers	Underberg (SA)		1	Hunter-gatherer
Nama	NAM	Khoe-San	Khoe (KhoeKhoe)	Windhoek (NM)		28	Herder
/Gui, //Gana and Kgalagari	GUG	Khoe-San	Khoe (Kalahari-Khoe)	Kutse Game reserve (BT)		22	Hunter-gatherer and Farmer
Naro *	NAR *	Khoe-San*	Khoe (Kalahari-Khoe)	Johannesburg (SA)	Ghanzi (BT)	2	Hunter-gatherer
Ju/’hoansi	JOH	Khoe-San	Ju	Tsumkwe (NM)		42	Hunter-gatherer
!Xun	XUN	Khoe-San	Ju	Omega camp (NM) and Schmidtsdrift (SA)	Menongue (AN)	49	Hunter-gatherer
Khwe	KWE	Khoe-San	Khoe (Kalahari-Khoe)	Omega camp (NM) and Schmidtsdrift (SA)	Caprivi Strip (NM)	18	Hunter-gatherer
Manyanga	DRC	Bantu-speaker	Bantu (central)	Luozi (DRC)		14	Farmer
Herero	HER	Bantu-speaker	Bantu (southwestern)	Windhoek (NM)		15	Farmer and Herder
Sotho, Tswana	SOT	Bantu-speaker	Bantu (southeastern)	Various (SA)		22	Farmer
Swazi *	SWZ *	Bantu-speaker*	Bantu (southeastern)	Chrissiesmeer (SA)		5	Farmer
Zulu, Xhosa	ZUX	Bantu-speaker	Bantu (southeastern)	Various (SA)		36	Farmer
Afrikaner	AFR	Non-African	Non-African	Various (SA)		21	Mixed
European	EUR	Non-African	Non-African	Various (SA)	Europe and Canada	11	Mixed
Indian	IND	Non-African	Non-African	Various (SA)		25	Mixed
**Total**						**538**	Mixed

AN – Angola.

BT – Botswana.

DRC – Democratic Republic of Congo.

NM – Namibia.

SA – South Africa.

* Small groups of individuals not included in group-wise analyses but included as individuals in network, tree and haplogroups assignments.

**Figure 1:**
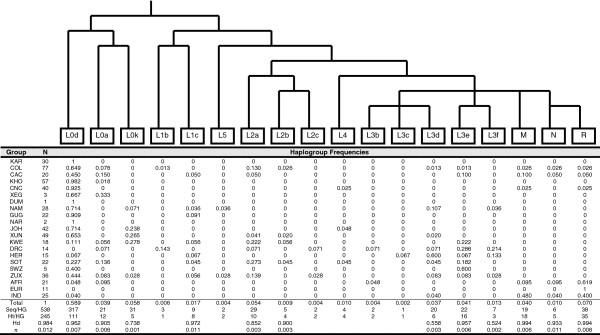
**Main mitochondrial DNA haplogroup assignments.** Mitochondrial DNA haplogroup tree with nomenclature according to Behar *et al.,*[12], listing haplogroup frequencies in the different populations in the study group. The number of sequences per haplogroup (Seq/HG), number of haplotypes per haplogroup (Ht/HG), Haplotype Diversities (Hd) and Nucleotide Diversities (π) in the different haplogroups are also indicated.

The 538 sequences were resolved into 18 haplogroups encompassing 245 haplotypes (Figure 1 and Additional file 2: Figure S2). Haplogroups other than L0d were found at very low frequencies in the total sample group (no other haplogroup was found at a frequency >7%). High frequencies of these non-L0d haplogroups were mostly seen in the comparative groups and not in the Khoe-San or sampled Coloured groups. L0d was the commonest haplogroup in the total sample (59%) and had high frequencies in all of the Khoe-San and Coloured groups ranging in frequency from 45% in the Cape Coloured to 100% in the Karretjie People (see [17] for further information on this population group).

**Figure 2:**
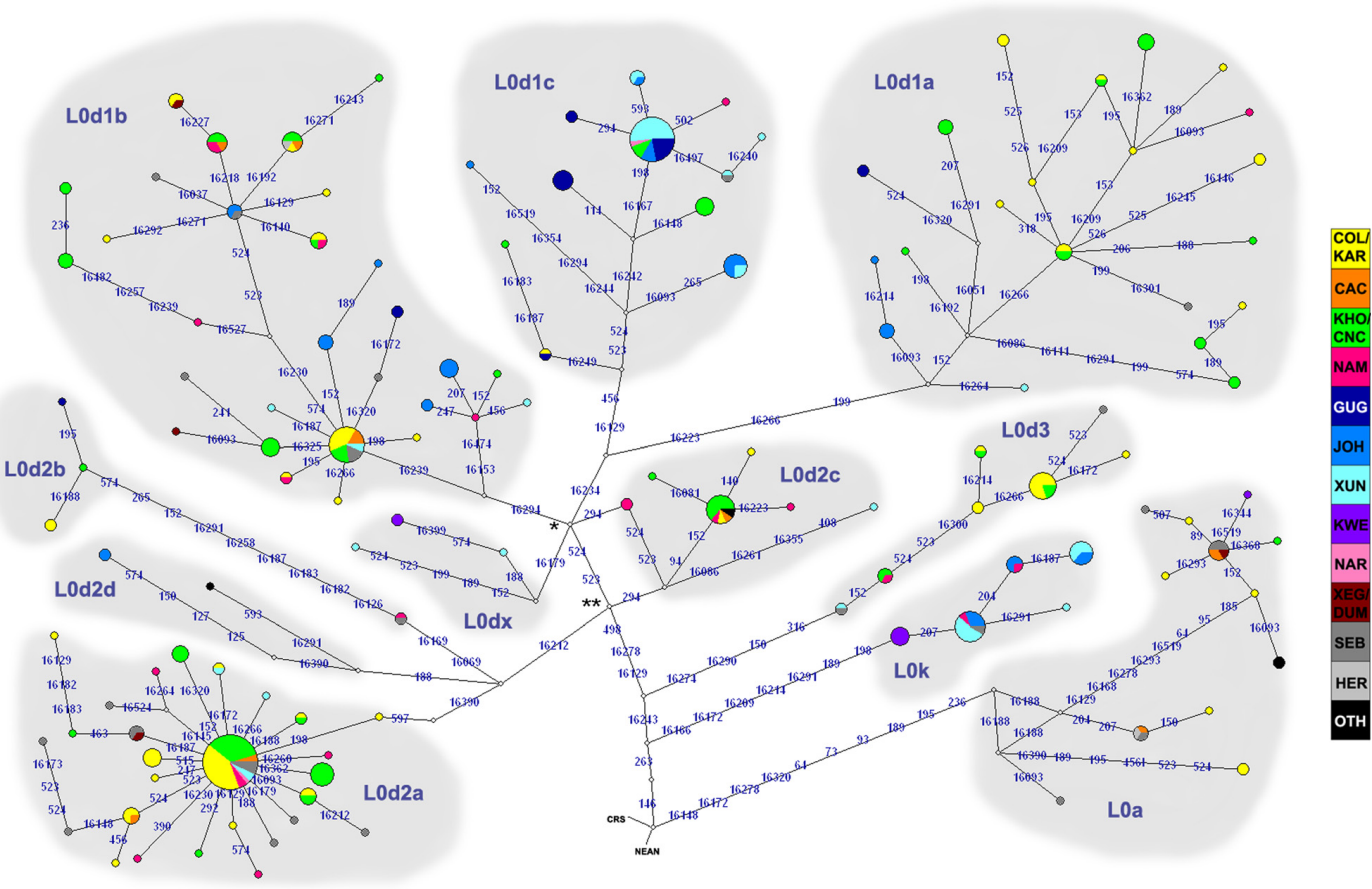
**Network of the L0 clade.** Median joining network representing L0 substructure in the different study populations. Stars indicate median vectors that are discussed in the text. CRS – Control Region Sequence, NEAN – Neanderthal – Root. Numbers indicate mutations according to HVS basepair number. Circles represent haplotypes and are proportional to the number of sequences represented. The color key indicates from which populations different haplotypes originated (Table 1). OTH (other) includes IND, AFR and EUR. SEB (southeastern Bantu-speakers) includes SOT, ZUX, SWZ.

The association of the HVS haplotypes within their main haplogroups was assessed using maximum likelihood trees (Additional file 2: Figures S3a and b) and parsimony based network analysis (Figure 2). Alignments and phylogenies were submitted to treeBASE (Access at: http://purl.org/phylo/treebase/phylows/study/TB2:S13870?format=html). Most haplogroups and sub-haplogroups grouped together on the tree; however, within the haplogroups L3, L4, M, N and R, the tree lacked structure. Thus, for certain haplogroups, the lack of variation in the HVS-I and II necessitate the use of coding region variation to indicate and direct the overall classification and structure of haplogroups (as was done using the minisequencing method).

### Evolutionary history of mtDNA haplotypes in haplogroups L0d/k

**Figure 3:**
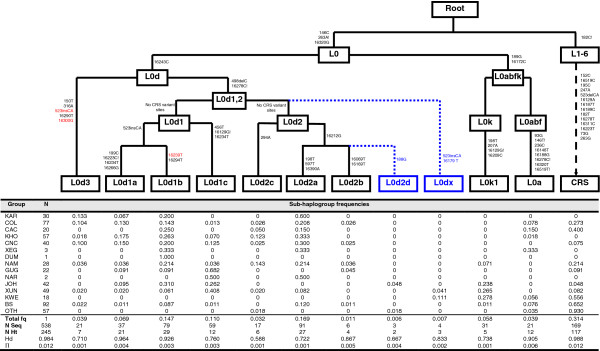
**Mitochondrial haplogroup assignments for the L0 clade.** L0d structure as published in Behar *et al*., [12] (black). Suggested changes are highlighted: In blue – Two new, previously unidentified clades. In red – Mutations suggested to be removed as clade defining mutations. The table summarizes the frequencies, Haplotype Diversities (Hd) and Nucleotide Diversities (π) of the various L0d subgroups as well as L0k1 and L0a. OTH – Other (includes IND, AFR and EUR, Table 1) and BS – Bantu-speakers (includes HER, SOT, ZUX, SWZ, Table 1).

MtDNA haplotypes within sub-haplogroups belonging to the L0 clade were clearly associated in networks and trees (Figure 2 and Additional file 2: Figure S3, respectively). The Khoe-San associated haplogroups L0d and L0k were distributed at different frequencies in the different Khoe-San groups (Figure 3 and Additional file 2: Figure S4). Coalescent times (Time to Most Recent Common Ancestors - TMRCA) for all the L0d/k subgroups, and times at which their lineages diverged from the other lineages, were calculated from the network (Table 2, Additional file 1: Table S2, and Additional file 2: Figure S5). Although several studies have estimated mtDNA mutation rates (Additional file 1: Table S2), we used the mutation rate suggested by Ward *et al*. [18] in the present study (this rate is very similar to the rate used by Soares *et al.,*[19] for the combined HVS I and II, see Additional file 3: Supplementary methods).

**Table 2:** TMRCA calculated for the L0d/k subgroups and comparative whole genome estimates

**Divergeance times**						
**Haplogroup**	**ρ**	**σ**	**Years **^**1**^	**SD **^**2**^	**Years **^**3**^	**CI**
L0d	10.8580	2.2635	96601	20138	152,384	12,698
L0d1a	7.1351	2.0865	63480	18563	34,921	6,349
L0d1b	4.6709	1.5502	41556	13792	34,921	6,349
L0d1c	6.7119	2.2906	59714	20379	53,175	7,936
L0d2a	3.8242	1.7380	34023	15463	64,287	8,730
L0d2b	8.5000	2.4777	75623	22044	64,287	8,730
L0d2c	3.2941	1.5519	29307	13807	64,287	8,730
L0d2d	5.0000	1.8559	44484	16512	-	-
L0d3	8.0000	2.6273	71174	23375	100,795	10,317
L0dx	4.0000	1.5411	35587	13711	-	-
L0k1 ^4^	8.5161	2.8144	75766	25039	142,860	11,905
**Coalescent times (TMRCA)**						
**Haplogroup**	**ρ**	**σ**	**Years **^**1**^	**SD **^**2**^	**Years **^**3**^	**CI**
L0d	9.8580	2.0307	87705	18067	100,795	10,317
L0d1	6.4286	1.2576	57194	11189	53,175	7,936
L0d2	4.8718	1.6199	43343	14412	64,287	8,730
L0d1a	4.1351	1.1634	36789	10351	18,254	6,349
L0d1b	3.6709	1.1845	32659	10538	16,667	6,349
L0d1c	4.7119	1.8019	41921	16031	23,810	5,555
L0d2a	1.8242	1.0143	16230	9024	8,730	3,174
L0d2b	6.5000	2.0344	57829	18100	-	-
L0d2c	2.2941	1.1867	20410	10558	20,635	5,555
L0d2d	4.0000	1.5635	35587	13910	-	-
L0d3	4.0000	1.7037	35587	15157	31,746	7,936
L0dx	3.0000	1.1726	26690	10432	-	-
L0k1 ^4^	1.5161	0.9596	13488	8538	39,683	8,730

^1^ Years are calculated using ρ [20] and multiplying with the mutation rate from Ward et al., [18].

^2^ Standard deviation (SD) are calculated from σ by multiplying with the specific mutation rate from Ward et al., [18].

^3^ Estimates based on whole mitochondrial genomes obtained from Behar et al., [12].

^4^ L0k estimates from present study only include L0k1, while the Behar et al., study include L0k1 and L0k2 haplotypes.

**Figure 4:**
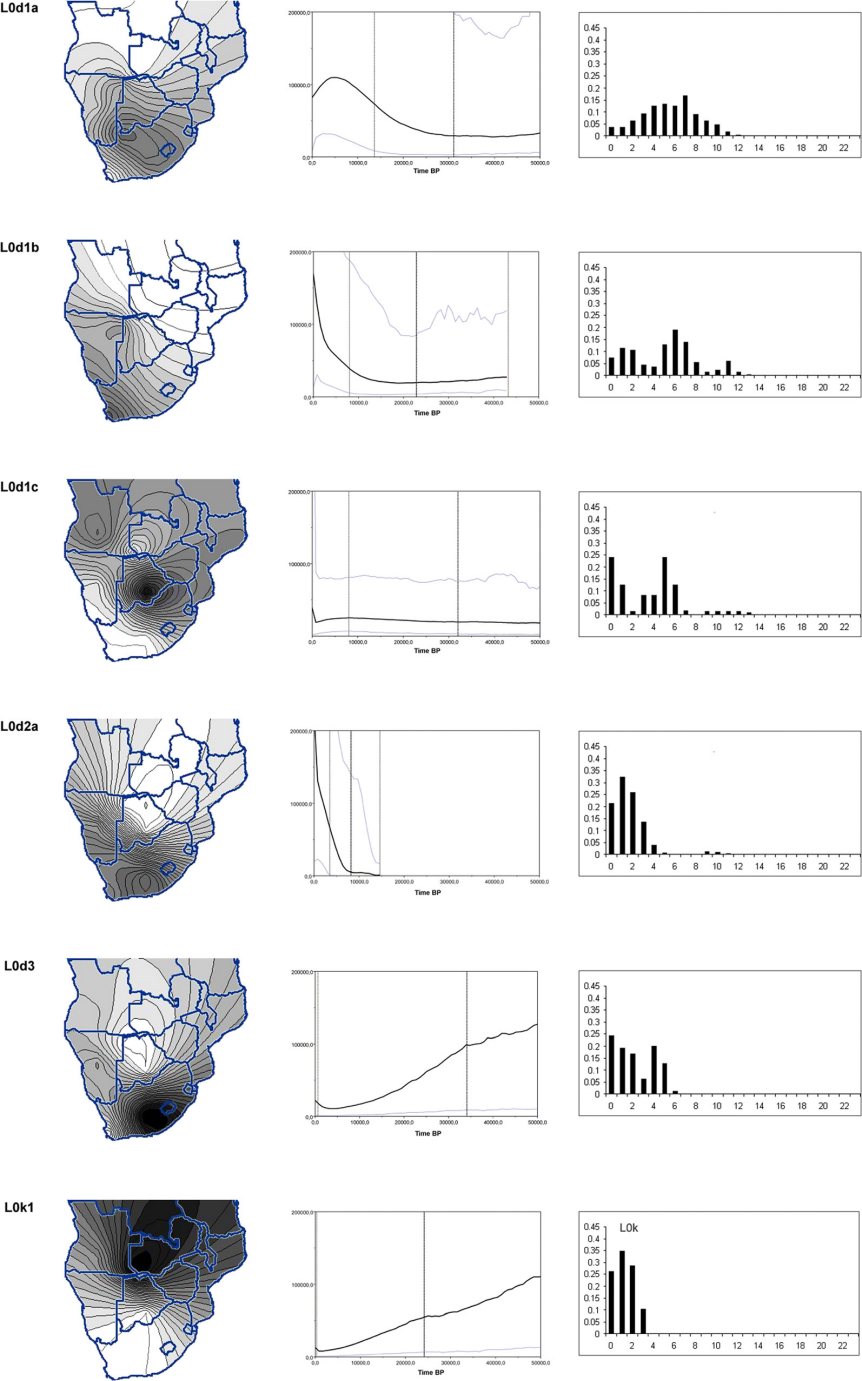
**Contour plots, Bayesian Skyline Plots and mismatch distributions of L0d/k haplogroups.** First Column: Contour plots indicating the frequency distributions of L0d/k subgroups. Color range is from 0 (white) to >60% (black) in increasing shades of gray. Second Column: Bayesian Skyline plots of haplogroups showing changes in *N*_*e*_ through time. *N*_*e*_ is represented on the Y-axis, while years ago are represented on the X-axis, with the present indicated by 0. L0d3 includes only L0d3 sequences from the present study (see Additional file 2: Figure S9 for inclusion of east African and Kuwait sequences). Black bold vertical lines indicate the coalescence date and the lighter vertical lines the 95% confidence intervals for the coalescence. Blue lines indicate the 95% confidence intervals for BSP lines (black). Third Column: Mismatch distributions of L0d/k sub-haplogroups.

Haplotypes within L0d/k found in the southern-San/Coloured/Khoe groups (KAR, COL, CAC, KHO, CNC, NAM) were clearly distinguished from those found in the San groups located north of them (GUG, JOH, XUN) (Additional file 2: Figure S4, see Table 1 for population labels). Contour plots, visualizing the geographic distributions of the L0d/k subgroups (Figure 4 and Additional file 2: Figure S6) showed that certain sub-haplogroups (L0d3, L0d2a, L0d1b) had higher frequencies in the south; others had a more intermediate and central distribution (L0d2c and L0d1a), while some had higher frequencies in the north (L0k1). L0d1c showed a bimodal distribution pattern (Figure 4), and the sub-haplogroup L0d1c1 (represented by the star-like expansion pattern within L0d1c in the network - Figure 2) had a distribution that differed from the remaining L0d1c haplotypes (Additional file 2: Figure S7). Remaining L0d1c sequences, after L0d1c1 sequences were removed (L0d1c-), had a unimodal distribution and high frequencies in the GUG group. The bimodal pattern of L0d1c is therefore the result of an expansion that gave rise to the L0d1c1 subgroup and elevated frequencies of L0d1c1 in the XUN. Since the L0d1c1 sequences coalesce ~2.47 ka (SD: 1.2 kilo years), the start of expansion should be around this time (coalescence of L0d1c1 sequences occur in the large central node).

Mismatch distributions were constructed for each sub-haplogroup, to further analyze the individual haplogroup histories and to test if they had notable expansions (Figure 4, Additional file 2: Figure S8 and Additional file 1: Table S3). All L0d sub-haplogroups except L0d2b, L0d2c and L0dx showed significant evidence of expansions. Sub-haplogroups L0d1a and L0d2a displayed high levels of significance and their mismatch distributions showed smooth unimodal distributions with low raggedness values, indicating that a single expansion occurred at some time in the past (indicated by the τ value). The τ values of all the L0d1 haplogroups were similar, with expansions dating to ~27 ka. L0d2a and L0k1 are associated with more recent expansions of ~6 ka. Both the M and R haplogroups are associated with older expansions ~40-50 ka, feasibly at the time when anatomically, cognitively and behaviorally modern Africans migrated to, and expanded in, Eurasia [21,22].

Caveats associated with the coalescence analysis employed in mismatch distributions are the assumption of a single, exponentially growing population and the large degrees of statistical uncertainty. Also, earlier population expansions can be obscured by recent population bottlenecks [23]. Mismatch distributions have been reported previously to have less ability to predict population expansions than neutrality test summary statistics such as Tajima’s D [24], Fu’s Fs [25] and the R2 statistic [26]. Diversity estimates, together with the neutrality tests for the L0d sub-haplogroups (and other haplogroups with >10 sequences) were also calculated and are provided in Table 3.

**Table 3:** Diversity statistics and neutrality tests of L0d/k subgroups and comparative haplogroups

**Group**	**N seq**	**N Ht**	**Hd**	**π**	**θ**_**S**_	**W-θ**_**S**_	**Ne**	**Tajima’s D**	**Tajima's D p-value**	**Fs**	**Fs p-value**	**R2**	**R2 p-value**
L0d	317	111	0.962	0.00732	0,01419	15.342	2730	−1.45547	0.028*	−33.984	<0.001***	0.0421	0.069
L0d1a	37	21	0.964	0.00436	0.00584	6.468	1151	−0.84168	0.210	−8.871	0.001**	0.0849	0.120
L0d1b	79	29	0.926	0.00279	0.00551	6.072	1081	−1.51856	0.040*	−17.250	<0.001***	0.0488	0.040*
L0d1c	59	12	0.760	0.00248	0.00388	4.305	766	−1.09831	0.136	−1.628	0.273	0.0714	0.186
L0d2a	91	27	0.722	0.00110	0.00463	5.116	910	−2.29659	<0.001***	−23.082	<0.001***	0.0240	0.005**
L0d2b	6	4	0.867	0.00458	0.00393	4.380	779	1.03370	0.864	1.229	0.701	0.2429	0.738
L0d2c	17	6	0.588	0.00129	0.00239	2.662	474	−1.65319	0.032*	−1.475	0.134	0.1135	0.128
L0d2d	3	2	0.667	0.00357	0.00359	4.000	712	na	na	na	na	na	na
L0d3	21	7	0.710	0.00121	0.00124	1.390	247	−0.08107	0.511	−1.287	0.170	0.1327	0.432
L0dx	4	3	0.833	0.00238	0.00244	2.727	485	na	na	na	na	na	na
L0k1	31	5	0.738	0.00110	0.00089	1.001	178	0.58176	0.742	−0.044	0.518	0.1522	0.721
L0a	21	12	0.905	0.00590	0.00526	5.837	1039	0.30935	0.676	−1.317	0.290	0.1475	0.725
L2a	29	10	0.852	0.00332	0.00367	4.074	725	−0.29380	0.430	−0.739	0.382	0.1153	0.472
L3d	20	6	0.558	0.00281	0.00253	2.819	502	0.41429	0.700	2.092	0.849	0.1506	0.662
L3e	22	16	0.957	0.00575	0.00545	6.035	1074	0.08399	0.601	−5.350	0.056	0.1345	0.611
M	19	18	0.994	0.00620	0.01340	14.592	2596	−2.17227	0.004**	−11.280	<0.001***	0.0462	<0.001***
R	38	35	0.994	0.00793	0.01702	19.326	3439	−1.93058	0.010**	−27.889	<0.001***	0.0466	<0.001***

* p-value < 0.05.

** p-value < 0.005.

*** p-value < 0.001.

Neutrality tests were used to detect deviations from the assumptions of neutrality and constant population size (significantly negative Tajima D and Fs values and significantly positive R2 values indicate population growth and/or positive selection). The R2 statistic is especially powerful when sample sizes are small (~10), and Fs have a greater ability to detect population expansions when sample sizes are large (~50) [26,27]. Haplogroups M and R, more commonly found outside of Africa, tested positive for population expansion using all three neutrality tests with highly significant p-values (Table 3). The L0d group as a whole had significant D and Fs values, but not for R2; the latter is a possible result of larger sample sizes [26]. Of the L0d sub-haplogroups, L0d2a had the highest significance when all three neutrality tests were used. L0d1b also attained significance in all three tests while L0d1a had a very significant Fs value, but did not reach significance in the Tajima’s D and R2 tests. Thus, collectively, mismatch distributions and neutrality tests unambiguously indicate a significant expansion in L0d2a and show strong support for expansions in L0d1a and L0d1b.

While neutrality tests are widely employed to test hypotheses of expansion events, recent improvements in coalescence inference methods led to increased accuracy, without the need to assume a single exponential growth curve [28,29]. One of these methods, Bayesian Skyline Plots (BSPs) [30], was employed to visualize the changes in *Ne* through time for each of the sub-haplogroups (Figure 4 and Additional file 2: Figure S9). It is important to note that apparent increase in *Ne* in a BSP could also be an indication of changes in population substructure. The L0d1a sub-haplogroup had an increase that started ~25 ka and a decrease around ~5 ka. L0d1b had an increase that started at ~15 ka and another recent increase. L0d1c appears to be associated with a constant population size over an extended period followed by a transient decrease around ~5 ka which then increased rapidly in the past 1 ka. Despite a shallow coalescence time, L0d2a showed a dramatic increase from ~8 ka onwards and a further recent increase. The L0d3 BSP profile that included east African and the Kuwait haplotypes (L0d3+) (from [10,12,31] as explained in the Methods section) showed a slow decline over an extended period followed by a recent increase in *Ne* (Additional file 2: Figure S9). The L0d3 profile that only included the southern African L0d3 haplotypes (L0d3) (from the current study) showed a more intense decline and an increase that started later than in the L0d3+ profile. L0k1 also showed a constant decline with a recent increase (Figure 4). The BSPs of all the L0d sub-haplogroups, except L0d1a, indicated a recent increase in *Ne* (Figure 4 and Additional file 2: Figure S9).

We also generated a BSP for the whole L0d haplogroup (Additional file 2: Figure S10), which shows an increase in *Ne* from ~50 ka and a more dramatic increase from ~5-10 ka.

### Genetic affinities of Khoe, San and Coloured groups with neighboring groups

**Figure 5:**
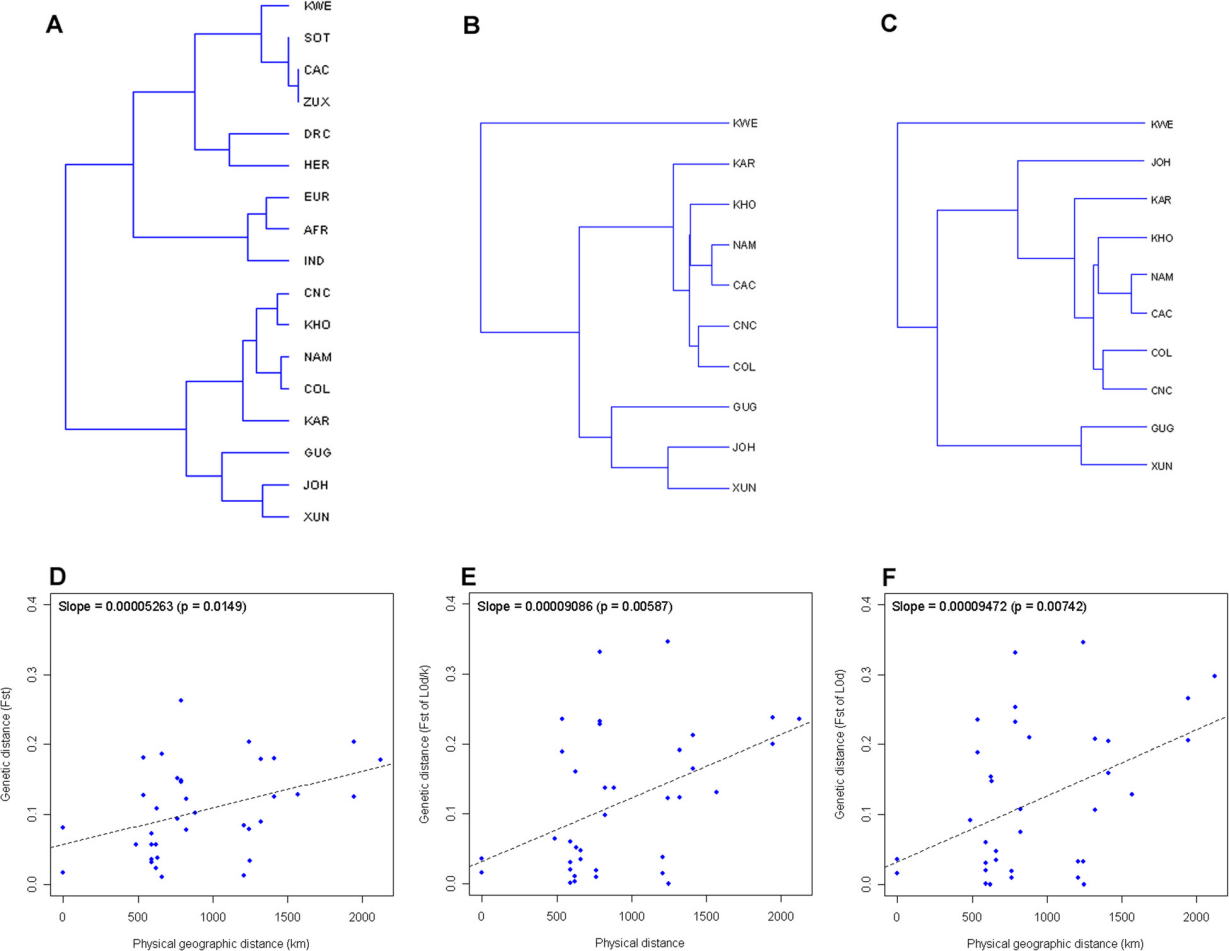
**Trees based on Fst and correlation of genetic distance with geographic distance between sample groups.** Trees representing mtDNA Fst values between different populations in the study group for **A**: all mtDNA sequences **B**: L0d/k sequences and **C**: L0d sequences. **D-F**: Pairwise comparisons between geographic distance (X-axis) and mtDNA Fst genetic distance (Y-axis), corresponding to trees in **A-C**.

To investigate the population group diversities and their relationship to each other and their neighbors, pairwise Fst’s between the different groups were calculated (Additional file 1: Table S4) and visualized in a UPGMA tree (Figure 5A). Populations with a considerable amount of Khoe-San ancestry, inferred from the presence of L0d/k haplogroups, grouped closely together in one clade, non-African populations grouped together, Bantu-speakers and populations with large proportions of admixture from Bantu-speakers (such as CAC and KWE) grouped together, and in another clade. Furthermore, the northern Ju-speaking San groups, JOH and XUN, grouped together with GUG on their own, while the southern Khoe-San and selected Coloured groups (CNC and KHO, COL and NAM, and KAR) grouped together.

To examine the evolutionary history of mtDNA haplogroups L0d/k further we removed all other mtDNA haplogroups from the groups and reanalyzed the data. The earlier observation (Figure 5A) of the distinction between the northern San groups (GUG, JOH, XUN) and southern Coloured and Khoe-San groups (KAR, KHO, NAM, CAC, CNC, COL) was once again clearly evident (Figure 5B). Also, the CAC group now clustered within the southern San-Khoe and Coloured groups. The KWE group, however, remains an outlier.

To examine how L0k could have influenced the population affinities of the northern and southern groups, we repeated the analysis with only L0d sequences (Figure 5C). The JOH group clustered with the southern Coloured and Khoe-San groups rather than with the GUG and XUN, but was still somewhat removed from them. This is not surprising since, from the L0d haplogroup frequencies (Figure 2 and Additional file 2: Figure S3b) it is apparent that JOH had much lower L0d1c frequencies than GUG and XUN, but higher L0d1b frequencies (frequent in southern groups). Other southern Coloured and Khoe-San groups still grouped tightly together in a cluster and the KWE group remained an outlier (but has to be viewed with caution because it is represented by only two L0d sequences).

To examine if the genetic distances (Fst) and geographic distances (km) between groups show any correlation within the Khoe-San-Coloured groups, the three pairwise Fst matrices were correlated with a geographic distance matrix (the KWE , Bantu-speakers and non-Africans were excluded for all three correlations). All three straight lines fitted to a graph of geographic distance vs. genetic distance, had significant positive slopes (Figure 5D-F). When non-L0d/k sequences were removed (Figure 5E), the slope was steeper compared to when all sequences were included (Figure 5D), and even steeper for L0d sequences only (Figure 5C). Mantel tests also confirmed the relationship between the genetic and geographic distances. For the correlation involving all sequences the correlation coefficient (r) was 0.402750 (p = 0.027), for L0d/k sequences, r = 0.449854 (p = 0.034) and for L0d sequences only, r = 0.439081 (p = 0.033). Both Mantel tests and linear regressions thus confirmed a positive relationship between geographic distance between groups and genetic distance. Maternal lineage-based population structure within Khoe-San groups is thus, at least in part, explained by geographic distance between the different groups. Furthermore, unequal amounts of admixture from Bantu-speakers and non-Africans into the various Khoe-San groups does not account for the observed pattern, instead admixture from these groups abated the relationship.

## Discussion

Two levels of mtDNA analysis, the first involving phylogeography, and the second population history, allowed us to evaluate how females have contributed in shaping the mtDNA pool of southern African Khoe-San and some Coloured populations. Like other studies before this [9,10,15,17,31,32], the high frequencies of haplogroup L0d among the Khoe, San and Coloured populations was found at varying frequencies in the groups we examined (Figure 1). Variation within L0d/k sub-haplogroups was found to be population specific and also showed differences in their geographic distributions (Figure 3). Using a combination of computational analysis and statistical tests we were able to evaluate the demographic histories of Khoe and San groups prior to, and after, contact with other groups in southern Africa. The genetic data, albeit only mtDNA, was used in conjunction with historical, linguistic, ethnographic and archaeological data, to better understand the prehistory of the Khoe and San.

It is generally accepted that southern Africa was occupied exclusively by Khoe and San people prior to the migration of Bantu-speakers to the region at ~2 ka [33]. From ~170 ka onwards the archaeological records at sites such as Pinnacle Point 13B [34], Blombos [35], Sibudu [36] and Border Cave [37], in southern Africa provide evidence for the presence of humans who started to think and behave in more complex ways than before. It is likely that the people associated with these cultural artifacts were ancestors of the present day Khoe and San; whether this assumption is true could potentially be resolved in future with ancient DNA studies. However, direct evidence of San-like hunter-gatherer cultural material in southern Africa has been reported as far back as ~40 ka [37], and autosomal DNA studies have shown that Khoe-San populations diverged from other human populations >100 ka [38-40].

### Notes on L0k and L0d topology

While many sequences (31) belonged to haplogroup L0k, they were represented by only five different haplotypes, all belonging to L0k1. L0k has two sub-haplogroups L0k1 and L0k2, that separated at ~40 ka [12]. As yet, there has been only one report of L0k2 in an individual from Yemen [12], while L0k1 was found exclusively in the San groups [9-12]. The L0k1 haplotypes of the current study coalesced by 13 ka and diverged from other sequences in L0 at 75 ka (Table 2).

Altogether, 317 sequences were resolved into haplogroup L0d (111 unique haplotypes). Haplotypes were grouped into the seven sub-haplogroups according to Behar *et al*., [12] and two extra previously unidentified sub-haplogroups (Figure 3). The L0d haplogroup was estimated to have a coalescence time of 87 ka (+/−18) and diverged from the other L0 groups by 96 ka (+/−20) (Table 2), a shallow time compared to whole genome studies [12].

Previously, L0d3 have been identified as the oldest L0d clade [12], and it also occurred as the earliest L0d branch on our tree and network (Figure 2 and Additional file 2: Figure S3b). The divergence from the other L0d sequences dated to 71 ka (+/−23) and L0d3 sequences coalesced at 35 ka (+/−15) (Table 3). Only two L0d3 sequences formed part of the whole genome study [12], one from a San individual and one from an individual from Kuwait (coalescence ~31 ka) and seven clade-defining mutations were identified [12]. In the present study, L0d3 is represented by seven haplotypes and based on data displayed in the network (Figure 2), it is suggested that 16300 G and 523delCA should be removed as clade defining mutations for L0d3 since earlier sequences in our network did not contain these mutations (Figure 2 and Figure 3).

Gonder *et al*. [31], did whole genome studies on a wide range of African sequences including the Platfontein !Xun and Khwe and the Sandawe from Tanzania. The !Xun/Khwe and Sandawe from that study were the only groups that contained L0d sequences (Table 1). When L0d sequences [31] were classified according to the nomenclature of [12], all the !Xun/Khwe L0d sequences were resolved into L0d1 or L0d2 (Table 1), while all the Sandawe L0d sequences fell into L0d3. When the L0d3 sequences from [31] and [12] were analyzed in a network, together with the L0d3 sequences from the present study, two separate groups formed; a Tanzania/Kuwait L0d3 group and a southern Africa L0d3 group (Additional file 2: Figure S11). It is suggested here that the Tanzanian/Kuwait sequences should be classified as an L0d3 sub-haplogroup, L0d3a, defined by the 16129 G-A, 16274 A-G! (reversion) and 16399 A-G mutations. The closest related haplotype in the southern Africa branch of L0d3 to the Tanzania/Kuwait branch occurred in the Karretjie People and Coloured groups from Colesberg. A haplotype found in the Karretjie People and Coloured groups was directly ancestral to the L0d3a branch (Additional file 2: Figure S11).

With the inclusion of the east African haplotypes, the whole L0d3 clade diverged from other L0d sequences ~83 ka, while all the L0d3 sequences coalesced at ~47 ka. The divergence of the L0d3a sub-haplogroup from the southern Africa haplotypes was dated at ~41 ka. L0d3a sequences coalesced at ~28 ka.

The L0d1,2 branch was separated from their ancestral node by two mutations, 498delC and a back-mutation, 16278C! (Figures 2 and 3). No HVS mutations differentiate between L0d1 and L0d2 (Figure 3). They are, however, defined by 5 and 6 coding region mutations, respectively [12]. When analyzing only the HVS, the branch separating L0d1 from L0d2, collapses. This can be seen in the network where all subgroups met at two central nodes (marked with * and ** in Figure 2). The absence of the 523insCA mutation (seen as an L0d1a-L0d1b defining mutation in Figure 3) separated the L0d2 sequences from the L0d1 sequences in the network.

The haplotype diversity for L0d1a (0.96) was the highest of all the L0d sub-haplogroups in the study (37 HVS sequences that included 21 haplotypes) (Figure 3). The 21 haplotypes converged by ~37 ka (Table 3 and Additional file 2: Figure S5), much older than the three L0d1a sequences in the whole genome study (at ~18 ka [12]).

The L0d1b clade was previously further defined by the 16239 T and 16294 T HVS changes (Figure 3) [12], however, a large subset of sequences from the present study, did not contain the 16239 mutation (Figure 2). It appears that 16239 should not be used as a clade defining mutation for L0d1b (Figure 3), but this will have to be assessed with whole genome sequencing. The coalescence times of the 29 HVS haplotypes from the present study and the four haplotypes employed in the whole genome study [12] were similar (Table 2).

The L0d1c haplogroup contained 59 L0d1c HVS sequences described by 12 unique haplotypes. The 12 haplotypes coalesced by ~42 ka and separated from L0d1a/b by ~60 ka (Table 2 and Additional file 2: Figure S5).

L0d2a was defined on the network by 597 T and 16390A (Figure 2). Previously, 198 T was also suggested as an L0d2a defining mutation [12], however, one of our L0d2a sequences did not contain the mutation. The 27 L0d2a HVS haplotypes of the present study had a TMRCA of ~16 ka (Table 3), coalescence analysis (applied in the BSP - Figure 4), however, dated the coalescence of L0d2a at ~8 ka, which is closer to the whole genome study estimate of ~9 ka [12] (Table 2).

The defining mutations of L0d2b were 16069 T and 16169 T. Four of the six sequences in L0d2b were separated by a very long branch (9 mutations) from the other two sequences and the L0d2b node had high coalescence and divergence times, higher than L0d3, which is inconsistent with previous classifications [12]. Three mutations on the long branch are highly reoccurring mutations and might decrease coalescence times if their weights are decreased, however, it remains highly likely that terminal nodes in this group were grouped incorrectly with other L0d1b haplotypes through network analysis. Although the terminal branches contain the L0d2b defining mutations, 16212, 16069 and 16169, whole genome sequences are necessary to establish whether they perhaps represent another L0d sub-haplogroup (Table 2 and Additional file 2: Figure S5). In the whole genome study [12], the one representative L0d1b sample, only contained the 16212, 16069 and 16169 mutations and not the additional mutations that defined the three terminal groups in the L0d2b clade on the network.

Three subgroups within L0d2 were previously identified; L0d2a, L0d2b and L0d2c [12]. The present study had representation across these three L0d2 haplogroups and also identified a fourth group; henceforth called L0d2d (Figure 3). L0d2d grouped with L0d2a/b and all three these groups were defined by the 16212 G mutation, while L0d2d was further defined by 188A-G (a reoccurring mutation), with an absence of the clade-defining mutations of L0d2a and L0d2b. Thus although L0d2d is suggested here as a new sub-haplogroup of L0d2, its position needs to be confirmed by whole genome sequencing.

The L0d2c sub-haplogroup consisted of 17 sequences (6 haplotypes), with a coalescence age of ~20 ka and divergence from L0d2abd at ~29 ka, the whole genome study had an older divergence date (~64 ka) [12] (Table 2 and Additional file 2: Figure S5). Only few HVS defining mutations for L0d2c exist (only 294a distinguishes the L0d2c from the L0d1,2 core haplotype), while several coding region mutations separate L0d2c from L0d2ab [12], which explains the differences in divergence times.

L0dx is the second new sub-haplogroup suggested in this paper, its exact position on the tree and network can only be affirmed through whole genome sequencing. It appears to be an L0d1 sub-group due to the presence of the 523insCA mutation; however, due to the instability of this length repeat mutation, we assigned the preliminary designation L0dx. The sub-haplogroup was further defined by the 16179 T mutation.

### Evolutionary history of mtDNA haplotypes in haplogroups L0d/k

All of the Khoe-San and sampled Coloured groups, with the exception of the Khwe, harbored L0d as the commonest haplogroup (Figure 1). L0d frequencies in the !Xun (65%) and the Khwe (11%) were similar to previously reported frequencies (Additional file 1: Table S1 and Additional file 2: Figure S4). If we assume L0d was the predominant Khoe-San associated haplogroup in the past, the BSP representation of change of *Ne* through time for the whole L0d clade can be seen as a representation of the population size change of all southern African Khoe-San predecessor population groups from ~100 ka till present (Additional file 2: Figure S10). Overall it appears if *Ne* of southern African Khoe-San groups increased from ~50-60 ka and even more so during the last 10 ka of the Holocene. Contextually, the first increase occurs during the later phases of the Middle Stone Age (MSA) and marine isotope stage (MIS) 3, and the second increase with the early Holocene or MIS 1 [41], both MIS 3 and 1 are considered phases of climate improvement after the cooler conditions often generalized for of MIS 4 and 2.

L0d sequences coalesce in the MSA phase informally known as the pre-Still Bay and would also include the Mossel Bay technocomplex [41,42]. They immediately precede a period of behavioral and cognitive florescence, linked with early evidence of modern or complex human behavior and cognition, expressed through material culture associated with the well-known Still Bay and Howiesons Poort technocomplexes [41,43,44]. In fact, finds indicating symbolically mediated behavior such as engraved ochre pieces and an ochre-processing workshop at ~100 ka [35,45,46] would seem to coincide with the coalescence of L0d haplogroups [12].

It appears that the subsequent expansion at ~50 ka gave rise to all the L0d1,2 sub-haplogroups (also see Additional file 2: Figure S5). Almost all the L0d1,2 sub haplogroups diverged roughly between 60 ka and 40 ka (Additional file 2: Figure S5). This was also the time-frame for the initial expansion of the L0d group as a whole (Additional file 2: Figure S10), and the various L0d1 and L0d2 subgroups could be seen as remnants of this expansion phase. This phase marks the early stages of the warmer MIS 3 and the end of the Howiesons Poort technocomplex at ~58 ka. Based on certain aspects of the archaeological record it is sometimes argued that so-called modern human behavior was ‘lost’ before becoming firmly established at ~40 ka, yet several lines of evidence, as well as theoretical reasoning, question this likelihood [41,47]. The period from ~58 ka to ~45 ka is known as the post-Howiesons Poort or Sibudu technocomplex [41,48], and at the name site in KwaZulu-Natal (South Africa) human behavior was clearly different, but no less complex than during the preceding Howiesons Poort and Still Bay phases [36,49,50]. Some of the behavioural trends, could indicate social and behavioural plasticity and complexity, and are not dissimilar to those observed among recent hunter-gatherers [51].

Subsequently, the L0d1 sub-haplogroups (especially L0d1a, L0d1b) showed signs of expansion during the transition of MSA to Later Stone Age (LSA). The final MSA (~20-40 ka) and the early LSA (~18-40 ka) phases overlap because of variability in stone tool assemblages and research to clarify this complicated transitional period is ongoing; at sites where all the archaeological phases are represented, we do not see dramatic breaks, but rather a gradual abandonment of MSA technologies in favor of transitional tool-making traditions culminating into LSA assemblages as a result of localized behavioral change and adaptation [42,51-53]. Thus far, the earliest art in southern Africa that can be associated with a shamanistic worldview was recorded at Apollo 11 in Namibia dated to ~30 ka [54]. This indicates that by this time people already had belief systems similar to those recorded for recent hunter-gatherers in the region [55]. The two haplogroups with a current southern distribution, L0d1a and L0d1b, had much stronger expansion signals during this transitional stage than the L0d1c haplogroup, which are currently associated with northern San groups. L0d1a had a growth signal that precedes the L0d1b growth phase by at least 10 ka. It was difficult to judge the start of expansion in L0d2a because of the shallow coalescence time of haplotypes. It might be that the L0d2a growth started in the same timeframe as L0d1b. L0d2a and L0d1b was the main groups in the southern populations and might share similar histories. The growth curve in L0d2a was, however, much steeper than in L0d1b.

L0d1a had a central distribution with the highest frequencies in the regions occupied by the ≠Khomani (Figure 3, Additional file 2: Figures S4 and S6). This haplogroup had low frequencies in most of the populations (<20%), but was geographically widespread and present in most groups (Figure 3 and Additional file 2: Figure S4). The BSP of L0d1a (Figure 4), showed a clear indication of an expansion that started between 25 ka and 30 ka, as well as a recent decline in population size from 3–4 ka to the present. The L0d1a network (Figure 2) showed a star-like expansion pattern associated with the southern groups. The central haplotype of the pattern was small and derivative sequences accumulated several mutations. This pattern is indicative of an older expansion in which the central haplotype declined over time and derivative haplotypes accumulated mutations. A mismatch distribution of L0d1a showed a smooth unimodal distribution with a low raggedness index suggesting a single expansion of the haplogroup by ~28 ka, as indicated by the τ value (Figure 4 and Additional file 1: Table S3). Of the three neutrality tests employed, only the Fs test showed a significant indication of an expansion (Table 3). The Fs statistic, however, is used widely and several studies showed it to be an accurate indicator of expansions [26,56].

The BSP of L0d1a furthermore showed a recent decline that started at ~4 ka, coinciding with the end of the Wilton technocomplex that started at ~8 ka, and is often considered a ‘classic’ LSA expression of complex, modern hunter-gatherer societies [57]. Archaeological site frequency can only be considered to vaguely reflect fluctuation in population sizes because of sampling bias. None the less, the archaeological record for southern Africa seems to indicate an increase in sites (and perhaps the general population size) during the last 4 ka. This increase was more prominent from ~2 ka onwards when herding was introduced to the region [33,58]. Reasons for the decline in L0d1a might be that groups carrying the L0d1a haplogroup in high frequencies were out-competed and displaced by other groups that expanded during this stage. It could be that L0d1a was the predominant group in hunter-gatherers who were displaced by pastoralist groups and/or lifeways. The latter might represent groups moving in from other areas, or drift effects of other haplogroups increasing within the same population. If hunter-gatherer groups in the past lived as small isolated metapopulations with limited amounts of gene-flow between them, the influence of genetic drift might have been more pronounced than in population groups with more gene-flow between groups [59].

L0d1b had a distribution that is concentrated in the south and declined towards the north (Figure 4). The Cape Coloured group had L0d1b as their most prevalent L0d haplogroup, while in the other southern groups it was the second most prevalent (Figure 3 and Additional file 2: Figure S4). Interestingly, it was also the most prevalent group in the Ju/’hoansi while frequencies in the other northern groups were lower (Figure 3 and Additional file 2: Figure S4). Published studies also found L0d1b to be the predominant haplogroup in the Ju/’hoansi [11] while occurring at low frequencies in the !Xun [9] (Additional file 1: Table S1).

The expansion dates for L0d1b indicated by the BSP are ~15 ka and 3 ka. Based on archaeological site frequency, the population density of the southern parts of South Africa, seems to have increased markedly from ~12 ka and particularly during the last 4 ka [33,58]. The expansion patterns seen for L0d1b thus loosely resemble the age estimates and increases in the archaeological sequence and site frequencies (Figure 4). The network also showed several star-like expansion patterns indicating that the haplogroup went through more than one phase of population growth (Figure 2), and the mismatch distribution indicated more than one expansion (a recent and an older expansion) (Figure 4). Additionally, all three neutrality tests significantly supported statistics that indicated expansion (Table 3).

L0d1c was completely absent or present at very low frequencies in the southern groups (Figure 4). It increased northwards, where it is the predominant L0d group (except in the Ju/’hoansi) (Figure 3 and Additional file 2: Figure S4). In published results, L0d1c was also the predominant group in the !Xun [9] and were undetected in the Ju/’hoansi [11] (Additional file 1: Table S1, Additional file 2: Figure S4).

A sub-clade of L0d1c, L0d1c1 was previously defined [12] and in our study most L0d1c sequences belonged to L0d1c1 (but only 6 of the 12 haplotypes) (Figure 3). L0d1c1 is represented by a large star-like pattern at the tip of the L0d1c network, which indicated a recent expansion (Figure 2). When the contour plot of L0d1c was split between the early L0d1c haplotypes and the L0d1c1 haplotypes, it was apparent that the early L0d1c haplotypes had their highest frequencies in the central /Gui+//Gana+Kgalagari group, but is almost absent in the !Xun (Additional file 2: Figure S7). L0d1c1 haplotypes, however, had their highest frequency in the !Xun. It therefore seems the L0d1c1 expansion is associated with the !Xun.

The low L0d1c frequency in the Ju/’hoansi was surprising given that they are geographically located between the !Xun and the /Gui+//Gana. The Ju/’hoansi and !Xun socio-economies, however, are vastly different; while the Ju/’hoansi continued to live as foragers, the !Xun adopted crop cultivation and herding from the local Ovambo population, with whom they have lived in close association for centuries [3,60]. This change in the !Xun lifeway could explain why the BSP for L0d1c show a decline of *Ne* concurrent with the introduction of pastoralism to their area, but then started to increase rapidly by 1 ka (Figure 4).

The L0d1c BSP plot (Figure 4) showed a slight increase in population size throughout the LSA from ~25 ka until ~5 ka. This increase, however, was not comparable to the dramatic increase seen in L0d1a and L0d1b as seen in the Model (SSD) p-value of the mismatch distribution (Additional file 1: Table S3) and BSPs (Figure 4). Furthermore all three neutrality tests rejected the expansion hypothesis (Table 3).

The L0d2a haplogroup had a distribution concentrated in the south (Figure 4), and it was the most prevalent L0d group in all the southern groups, except the Cape Coloured where it was the second most prevalent. All the southern groups had either L0d2a or L0d1b as their most prevalent and second most prevalent groups (Figure 3 and Additional file 2: Figure S4). L0d2a was absent in most northern groups and at low frequencies in the !Xun. L0d2a was the L0d group that had the highest incorporation into the Bantu-speaking groups (Figure 3 and Additional file 2: Figure S4).

L0d2a formed a large star-like expansion pattern that is indicative of a massive recent expansion in the population groups represented in the haplogroup (Figure 2). All three neutrality tests detected an expansion in L0d2a with the highest associated significance of all the L0d/k subgroups (Table 3). The mismatch distribution did not reject an expansion hypothesis and the mismatch graph shows a smooth unimodal curve that indicated a recent expansion (Figure 4 and Additional file 1: Table S3). The τ value dated the expansion to ~7 ka (Additional file 1: Table S3). Looking at the BSP (Figure 4) one can see an immediate dramatic increase in L0d2a *Ne* from the coalescence date (~8 ka) onwards until present. A further, recent expansion (at ~1 ka) was also evident. In the span of 8 ka the *Ne* increased >20 times (Figure 4), these data are consistent with apparent increases in archaeological site frequencies in South Africa and Lesotho [41]. The coalescence age can be archaeologically linked to the start of the Wilton technocomplex that is widely distributed across the South African-Lesotho landscape [41] and represents a microlithic industry.

Most haplogroups show abrupt increases in population sizes around the time of the introduction of pastoralism (Figure 4 and Additional file 2: Figure S9). This could be due to population increases coupled to the adoption of pastoralist practices or population increases due to indirect benefits gained from surrounding pastoralist groups. Alternatively (or additionally), the increases in the BSPs can be an indication of changes in population sub-structure during these times, which could be expected with the drastic social changes linked to a shift in subsistence practices. While L0d2a had a more recent expansion phase that correlated with the introduction of pastoralism, the major part of the L0d2a expansion phase predated the introduction of sheep and/or goats into the southern regions. This rapid increase might be part of the increase noted in the archaeological record that occurred from ~12 ka onwards. This was, however, before the coalescence time indicated on the BSP (Figure 4). From archaeological and paleo-environmental studies we know that the period between 10 ka and 5 ka is associated with the reach of maximum temperatures after the Last Glacial Maximum (LGM) and the completion of the rise in sea levels. It might be that these events concentrated populations and increased social networking, which led to the spread of technologies between groups in the south. Perhaps inducing development of microlithic technocomplexes such as the Wilton technocomplex. Further expansions into new habitats and elaboration of material culture and technology, are noted in the archaeological record from ~4 ka onwards [41].

In contrast to the above-mentioned haplogroups, L0d3, showed no evidence of expansions associated with the LSA to MSA transition, yet, it also had southern distribution. Drift effects could cause this haplogroup to decrease while other haplogroups in the same populations increased. Another explanation could be that this haplotype was not subjected to similar conditions as the other L0d haplotypes during the early and middle LSA and thus might have only been introduced to these territories subsequent to these phases. During the last 4–2 ka, however, the expansion phases of the L0d3 haplogroup correlates with the introduction of pastoralism in eastern and southern Africa respectively, so that we suggest that the populations that carried it either adopted or benefited from herding.

L0d3 was present in the southern Khoe-San and Coloured groups but almost absent in the northern groups (only one !Xun individual was assigned to L0d3) (Figure 3 and Additional file 2: Figure S4). Although L0d3 had low frequencies compared to the other L0d subgroups, in all the southern groups, its distribution showed a south–north cline. When results from three previous studies [9,10,31] are combined with the present study, a group of 225 !Xun, Khwe and Ju/’hoansi were screened and only one !Xun individual (from the present study) contained an L0d3 sequence. This indicates an extremely low incidence of L0d3 in the northern San groups.

Tishkoff *et al*., discussed the possibility that the linguistic connection between the Sandawe and Kalahari-Khoe languages [7] was associated with the L0d genetic connection [10]. They concluded that the maternal genetic connection between the two groups was very deep (>15 ka) and it was unlikely that linguistic traces can be detected that far back. From the present study it seems unlikely that a linguistic connection between the Sandawe and the Khoe-speaking San groups in Botswana was associated with the L0d3 lineage. Although L0d3 was the exclusive L0d lineage in the Sandawe, it was almost completely absent in the northern and central Khoisan-speaking groups. In contrast to this absence in the northern and central groups, the southern groups contained higher L0d3 frequencies and the frequencies were the highest is in the Karretjie People from Colesberg. Furthermore L0d3 sequences were detected in a Bantu-speaking individual from Mozambique [61,62] as well as an individual from northern Kenya [63] and an individual from Kuwait [12]. This suggests an L0d3 spread along the eastern part of Africa, forming a connection between the southeastern Khoe-San groups and the Tanzanian Sandawe rather than between the northwestern Khoe-San groups and the Sandawe as the linguistic connection seems to suggest.

BSP analysis (Figure 4 and Additional file 2: Figure S9) indicated that the southern African L0d3 shows a steady decline from the coalescence point onwards with a sharp increase starting at ~2 ka. When the east African sequences were included, the decrease was not as severe and the recent expansion started earlier (at ~4 ka) (Additional file 2: Figure S9). The recent expansion phases of the haplogroup correlates with the introduction of pastoralism in east Africa and southern Africa, respectively. It is therefore likely that the populations that carried L0d3 either adopted the herding economy or benefited from it. In South Africa the introduction of pastoralism is associated with the ceramic final LSA, the only Stone Age phase that also has ceramics and domesticated animal remains in its assemblages [41,57], and although a few such sites date to just before 2 ka [64], most post-date this age. It is notable that not all archaeological assemblages post-dating ~2 ka has ceramics or evidence of domesticated animals. From the archaeological record it seems that *bona fide* hunter-gatherer groups have co-inhabited the landscape with early herders [65].

Most other haplogroups also showed expansions during the time of the introduction of pastoralism to the southern parts of Africa. An exception was haplogroup L0d1a that showed a decrease during this time. This decrease could be due to drift or could indicate that the groups carrying L0d1a in high frequencies were negatively affected by this stage. It is historically known that when pastoralists enter a territory, hunter-gatherers are displaced to fringe areas, which is unsuitable for stock-keeping. This would impact on the success of the hunter-gatherer population measured through population growth. From this it is deduced that carriers of L0d1a could have been populations that continued their hunter-gatherer lifeway and did not adopt pastoralism or enter in to favorable relationships with pastoralists. Initially L0d1c also started to decline, similar to L0d1a, but then started to increase. This turnaround might be associated with the recent partial adoption of pastoralism practices in the !Xun, through beneficial contracts with Bantu-speakers [3].

Frequencies of some L0d sub-haplogroups (L0d2b, L0d2c, L0d2d, L0dx) were too low to extract any information regarding their history. L0d2b was detected at very low frequencies in the present study (six sequences that represents four haplotypes) (Figure 3 and Additional file 2: Figure S4). It was also not detected previously in the !Xun, Khwe or Ju/’hoansi [9,11]. L0d2c were found at lower frequencies overall (Figure 3 and Additional file 2: Figure S4) with the highest frequencies in the ≠Khomani and Nama (in other populations it was <5% of L0d/k haplogroups). A star-like expansion pattern in the network seemed to be associated with the ≠Khomani group (Figure 2) but expansion hypothesis was rejected in the mismatch distribution and only one neutrality test detected evidence for an expansion (Additional file 1: Table S3 and 3). The star-like pattern of a very recent expansion in the network could, however, be observed in the mismatch graph (Additional file 2: Figure S8). This recent expansion in L0d2c correlated temporally with the recent expansions in other haplogroups and is likely to be associated with the introduction of pastoralism. Haplotypes that can be classified as L0d2d were reported previously in Bantu-speakers [61,62] and in the !Xun/Khwe [10]. In the present study L0d2d was confined to the Ju/’hoansi, where it represented 5% of L0d/k haplogroups (Figure 3 and Additional file 2: Figure S4). L0dx was found only in the two northern-most groups, Khwe (11%) and !Xun (4%) (Figure 3 and Additional file 2: Figure S4). L0dx was the only L0d haplogroup found in the Khwe. In the study of Chen *et al*., L0dx was found at similar frequencies in the !Xun (6%) but at much higher frequency in the Khwe (42%), where it also was the only L0d haplogroup [9]. From the network (Figure 2) it is evident that the Khwe all belong to one haplotype and a !Xun haplotype was ancestral to the Khwe haplotype. It therefore seemed that L0dx was an original !Xun haplotype and, through gene-flow, was assimilated into the Khwe. This was the reverse situation as was seen for L0k. The frequencies of L0dx was, however, very low and more L0dx haplotypes need to be sampled before any deductions can be made with certainty (the !Xun and Khwe L0dx haplotypes reported in Chen *et al*., [9] did not include the 16399 and 574 regions (Figures 2 and 3) and therefore could not be resolved further).

On a geological timescale, the divergence of the L0k haplogroup (75 ka) coincides with the beginning of marine isotope stage (MIS) 4 that is associated with a cold to very cold phase, not dissimilar to the LGM or MIS 2 (at ~32-13 ka), which could have contributed to the isolation of population groups and the divergence L0k lineage form other mtDNA lineages. In the present study, L0k was only found in the northern Khoe-San groups (Figure 1 and Additional file 2: Figure S4). The L0k1 haplotypes found in the Nama (low frequencies) is likely the result of recent gene flow from the San people of northern Namibia (such as the Ju/’hoansi and !Xun), since the Nama originated in the Northern Cape Province (SA), more or less where the ≠Khomani groups are located today, and only recently moved into central and northern Namibia [3]. The frequencies of L0k in the !Xun and Khwe from this study (27%, 28%) were comparable with previous studies (Additional file 2: Figure S4 and Additional file 1: Table S1). Our results for the Ju/’hoansi (24% L0k and 71% L0d), differed somewhat from previous findings for this population (4% L0k1 and 96% L0d [11]). The two Ju/’hoansi groups were, however, not from the same locations, the group of the present study was sampled in Tsumkwe (Namibia) and the previously published group was sampled in Botswana (Dobe) as well as in Namibia. It is possible that the Tsumkwe group had more gene-flow with the neighboring !Xun groups.

Since previous studies only reported on the mtDNA haplogroup frequencies of the three northern San groups (!Xun, Khwe and Ju/’hoansi) the low frequency of L0k1 in the Khoe and the absence in the southern San and Coloured groups have never been noted before. Salas *et al*., however, noted its complete lack in southeastern-Bantu-speakers contrasting with L0d [61]. Previously, it was thought that the history of L0d and L0k is closely intertwined and synonymous with Khoe-San history [12,61,66]. From the present study it was clear that, although all groups in this study with Khoe-San ancestry had L0d in common, L0k was only associated with the northern Khoe-San groups (Figures 1. 2, 3, Additional file 2: Figure S4).

The history of the L0k1 haplogroup might be closely tied up with the Khwe rather than the rest of the San groups. It was the haplogroup with the highest frequency in the Khwe, whereas in the other northern San groups (!Xun and Ju/’hoansi) it was secondary to L0d groups (Figures 1 and 3) and might have been introduced to these groups through gene flow with the Khwe and related groups. The low L0k1 haplogroup diversities suggest only few founders (Figure 1). In the network and tree (Figure 2 and Additional file 2: Figure S3b) it could be seen that all the Khwe sequences belonged to one haplotype and that the Khwe haplotype was ancestral to the haplotypes observed in the !Xun, Ju/’hoansi and Nama. This suggests that L0k1 was originally a Khwe haplogroup, and spread to the other northern San groups from where it diverged further. In the study by Chen *et al*., L0k1 also was the predominant haplogroup in the Khwe (Additional file 1: Table S1) [9]. Furthermore, all seven L0k1 sequences identified in the Khwe by Chen *et al*., was identical to the L0k1 Khwe haplotype of the present study, whereas ten of the eleven L0k1 sequences in the !Xun was derived from the ancestral Khwe haplotype (one !Xun sequence had the ancestral Khwe haplotype) [9].

It is unclear where the Khwe originally came from. They could be Khoe-San groups with extensive Bantu-speaking admixture, Bantu-speakers that lost their cattle, another pastoralist population closely related to Bantu-speakers who occupied the region before the Bantu-speaker expansions, or perhaps a mixture of various refugee groups driven from the grazing grounds into the Okavango swamps [67]. Genetic results from this study indicate that the maternal lineages of the Khwe showed contributions from southeastern Bantu-speakers and Khoe-San (Additional file 2: Figures S2 and S4). In addition they might have had a unique contribution from an unknown pastoralist or hunter-gatherer population that carried high frequencies of the L0k1 maternal lineage, whose identity has since been lost. The discovery of the L0k2 haplogroup in an individual from Yemen [12] suggests that the L0k haplogroups might have had an extensive spread in prehistoric Africa, but remnants of the haplogroup in other populations have been lost due to drift or has not been detected due to insufficient sampling. Alternatively, it could be the result of a more recent introduction as part of the African Diaspora, for example the slave trade.

Ancient hunter-gatherer populations that lived northeast of the Khoe-San groups, prior to the spread of the Bantu-speaking-groups might have been carriers of L0k haplogroups. These groups might have had linguistic and genetic connections with both the Khoe-San and East and Central African hunter-gatherers. An ideal candidate for such a group might be the Pygmy groups that lived north of the Khoe-San before the Bantu-speaker expansions. MtDNA studies, however, found no L0d or L0k haplogroups in the Pygmy groups of central Africa that has been studied thus far [68]. Currently Pygmy groups are mostly assigned to a specific L1c haplogroup. The remnants of the southern Ba-Twa Pygmies have, however, not been studied genetically and it is possible that they might contain maternal genetic connections to the Khwe.

In addition to the L0d/k groups in the Khoe-San and Coloured groups there were also a contribution of haplogroups resulting from admixture from Bantu-speakers and Eurasian groups (Figure 1 and Additional file 2: Figure S2). From the groups that represent the people with southern Khoe-San ancestry, the Karretjie People and ≠Khomani groups had almost exclusive L0d maternal lines, while the Coloured group from the Northern Cape also had very high percentages of L0d. The Coloured group with the largest proportion of admixture was the sample group from Wellington, with 20% Eurasian admixture and 35% Bantu-speaking admixture. The Colesberg Coloured group also had large proportions of Bantu-speaking (27%) and Eurasian (8%) admixture. The Coloured group from the Northern Cape had 5% Eurasian admixture and 2.5% Bantu-speaking admixture. The three Coloured groups were the only groups with Eurasian admixture, the admixture in the remaining Khoe-San groups were due to gene-flow with the Bantu-speaking groups (Figure 1 and Additional file 2: Figure S2).

The Khwe group had the largest input from Bantu-speaking groups (61%) with two of the most common southeastern Bantu-speaking associated haplogroups, L2a and L3e constituting the largest part (22% each) (Figure 1 and Additional file 2: Figure S2). The Nama had 21.5% Bantu-speaker admixture and in this case the Bantu-speaker-admixture was indicative of admixture with southwestern Bantu-speakers (L1c, L3d and L3f haplogroups). The remaining San groups had < 10% Bantu-speaker admixture (Figure 1 and Additional file 2: Figure S2).

### Genetic affinities of Khoe, San and Coloured groups with neighboring groups

Using the maternally transmitted mtDNA marker, the affinities between the present day Khoe-San and some Coloured groups were examined. Since the associated haplogroups of the Bantu-speakers and non-African groups are very distinct from those commonly found in the Khoe-San, admixture from these populations will have a great influence in the resultant tree that represents relationships between the different population groups. This can be seen in Figure 5A where the Bantu-speaking admixture in the Cape Coloured and Khwe groups cause them to group within the Bantu-speaking-clade. While an inclusive comparison is representative of the current genetic composition of the groups studied, it should not be used to make inferences about Khoe-San history and Khoe-San group relations before the expansions of Bantu-speakers and the influx of non-Africans.

In an attempt to infer group relations between the Khoe-San and the Khoe-San ancestors of some Coloured groups that existed before the influx of other groups, haplogroups associated with Bantu-speakers as well as non-African haplogroups were removed from the Khoe-San and sampled Coloured groups. Remaining Khoe-San associated haplogroups (L0d and L0k) were then used to infer relationships that might have existed in the past between the Khoe-San groups. The resulting tree (Figure 5B) showed that the southern groups were closely associated with each other while the northern groups are separate from the southern groups. The Khwe was different from all the groups. There was an association between geographic and genetic distance (Figure 5B), and it was stronger than the situation where Bantu-speaking and non-African haplogroups were included (Figure 5A). Due to the possibility that L0k was not part of the original Khoe-San haplogroup pool but rather introduced by other hunter-gatherer groups that were displaced (such as previously discussed for the Khwe) or because of the Bantu-speaker expansions, L0k was also removed and only L0d based group relations was tested (Figure 5C). Interestingly, the Ju/’hoansi group moved closer to the southern groups due to the higher frequency of L0d1b and the lower frequency of L0d1c. The correlation between physical and genetic distance remained for the L0d based group comparison. Using the present day distribution of haplogroup lineages within a population group as proxy for historical relations is debatable, since group memberships are, and have been, fluid and might be more so for certain population groups than others. Furthermore, inferences based on one locus, such as mtDNA, is very susceptible to the effects of genetic drift on lineages. Certain major cultural shifts that occurred in the past, such as the adoption/introduction of pastoralism by Khoe groups might also have had a significant impact on haplogroup distribution patterns.

## Conclusion

The following deductions can be made about the maternal line genetic composition of the population groups included in the study. First, various levels of admixture from both Bantu-speakers and non-African groups are present in the different Khoe-San and some Coloured groups. Secondly, the Khwe group is different from the other Khoe-San and Coloured groups and might represent remnants of another extinct hunter-gatherer group that were displaced by the Bantu-speaking expansions and became associated with the San. Thirdly, there is a distance-based genetic relationship between the remaining Khoe-San groups. Fourth, the haplogroup distribution between the southern and northern groups is different and the pastoralist Khoe group, the Nama, clusters with, and is similar to, the southern groups.

Overall this study presents new insights into how mtDNA haplogroups and their distribution among southern African populations were used to reconstruct the demographic histories of Khoe-San and selected Coloured populations. The juxtaposition of archeological and linguistic data, where possible, in conjunction with the genetic data, provided a broad context for reconstructing the human past of the southern African region.

## Methods

### Subjects

DNA samples from 538 unrelated individuals were collected with the subjects’ informed consent, and the project was approved by the Human Research Ethics Committee (Medical) at the University of the Witwatersrand, Johannesburg (Protocol Number: M050902), the Working Group of Indigenous Minorities in Southern Africa (WIMSA) and the South African San Council. During sample collection, information regarding birthplace, relationship to other volunteers, home language and ethnic group were recorded. An interpreter was used during sample collections where the volunteers did not speak English or Afrikaans as a first or second language. A description of sample groups, group codes, place of sampling and origin, linguistic grouping and number of individuals are outlined in Table 1 and a map with sample locations is given in Additional file 2: Figure S1.

### DNA extraction

DNA was extracted from EDTA-blood using the salting-out method [69] and from buccal swabs using the PureGene® Genomic DNA Purification Kit (Gentra Systems) according to manufacturer’s instructions.

### MtDNA typing

MtDNA types were assigned to macro-haplogroups L0-L6, M, N and R using a minisequencing method [70]. The mtDNA control region was amplified and sequenced according to previously published methods [71] with some modification [70]. Sequence data were obtained for hyper variable segments I (HVS I; nucleotide positions 15997–16569) and HVS II (nucleotide positions 57–607). The base calls at positions 16184–16193 were ascertained by manual inspection of electropherograms and where there was any uncertainty about a mutation, the sequence was repeated with the reverse primer. Variation in the HVS-II 303–315 repeat track were not considered or reported in any of the analyses. Insertions in the poly C repeat track at position 568–573 where always taken as a 1 bp C insertion. When there was uncertainty whether a mutation in the 16184–16193 region was the result of a substitution or an in-del, the mutation was always treated as a substitution. All other regions were considered albeit some regions were sometimes differentially weighted as outlined in the individual analysis descriptions. Insertions or deletions were treated as a fifth state. Sequences were aligned using the Clustal W algorithm [72] implemented in BioEdit v.7.0.5.3 [73]. All sequences were compared to the revised Cambridge reference sequence [74] to establish the variant positions and were assigned to haplogroups according to the proposed nomenclature [12]. Combined HVS I and II sequences were used in further analyses. All sequences were submitted to Genbank (Accession numbers KC004766 - KC005303 for HVS I and KC004228 - KC004765 for HVS II sequences).

### Comparative data

Sequences from other sources included a Neanderthal sequence (Genbank accession number: NC_011137) [75], the revised Cambridge reference sequence [74] and additional published L0d3 sequences [10,12,31]. Sequences from [31] and [10] had overlap in some of the subjects and only one of the two in each case was selected. Problems with Genbank submissions from [31] were mentioned previously [17,76]. A complete description on sequences included from these two publications are found in [17] (especially note the explanation regarding isolate TZSW084).

### Computational analyses

Full details and parameters of computational analyses are included in the supplementary methods section (Additional file 3). The analyses included a maximum likelihood tree of all unique haplotypes; and furthermore, for L0d and L0k haplogroups and sub-haplogroups: a Median Joining network, TMRCA calculations, Mismatch distributions, isofrequency plots of the geographic distribution, Bayesian Skyline Plots (BSP) and various summary statistics and neutrality tests. For the population groups in the study, population pairwise Fst’s were calculated, visualized as trees and compared to geographic distances between populations using linear regressions and Mantel tests (groups with N<10 were excluded from the population analyses – Table 1).

## Competing interests

The authors have no financial or other competing interests to declare.

## Authors’ contributions

CMS and HS conceived of the study and participated in the sample collections. CMS carried out the molecular laboratory work, sequence processing and downstream computational and statistical analyses. CMS drafted and wrote the manuscript. HS participated in the design and coordination of the study and commented on the manuscript. ML contributed archaeological and marine isotope contexts. All authors read and approved the final manuscript.

## Supplementary Material

Additional file 1: Table S1. Published mtDNA haplogroup and sub-haplogroup frequencies in San populations. **Table S2.** TMRCA calculated for the L0d/k subgroups. Four different mutation rates are applied. **Table S3.** Mismatch distribution statistics. **Table S4.** Mitochondrial population pairwise Fst values (All sequences). (DOC 149 kb) Click here for file

Additional file 2: Figure S1. Map indicating the place of origin for the Coloured, Khoe and San individuals who participated in the study. Labels and further information are available in Table 1. Bantu-speakers and people of non-African descent were sampled from various locations in South Africa and are not indicated on the map. **Figure S2.** Bar plot of percentage mtDNA haplogroup assignment in study populations. **Figure S3a.** Maximum likelihood tree representing the substructure of L1 to L5. A Neanderthal sequence forms the outgroup. Branch support (%) was calculated through aLRT. **Figure S3b.** Maximum likelihood tree showing the relationships of the different mtDNA haplotypes within haplogroup L0. A Neanderthal sequence forms the outgroup. Branch support (%) was calculated through aLRT. **Figure S4.** Bar plot of percentage L0d/k sub-haplogroup assignment in study populations. Published comparative data is according to Table S1. **Figure S5.** Graphic representation of coalescent times and times of divergence of the mtDNA sub-haplogroups of L0d and L0k (according to Table 2). The mutation rate estimated by Ward *et al.,* (1991) was used in these estimates. **Figure S6.** Contour plots indicating the frequency distributions of L0d/k subgroups. **Figure S7.** Contour plots of L0d1c split into two subgroups, L0d1c1 and the remaining L0d1c sequences (L0d1c-). **Figure S8.** Mismatch distributions of L0d/k sub-haplogroups and comparative groups. # expansion hypothesis rejected (95% CI overlap). **Figure S9.** Bayesian Skyline plots of L0d haplogroups showing changes in *N*_*e*_ through time. *N*_*e*_ is represented on the Y-axis, while years before present are represented on the X-axis, with the present indicated by 0. L0d3+ is L0d3 including the east African and Kuwait sequences. L0d3- includes only L0d3 sequences from the present study. The black bold vertical lines indicate the coalescence date and the lighter vertical lines the 95% confidence intervals for the coalescence. The blue lines indicate the 95% confidence intervals for the plot-lines. **Figure S10.** Bayesian Skyline plots of all L0d haplotypes across all population groups, showing changes in *N*_*e*_ through time. *N*_*e*_ is represented on the Y-axis, while years before present are represented on the X-axis, with the present indicated by 0. The black bold vertical lines indicate the coalescence date and the lighter vertical lines the 95% confidence intervals for the coalescence. The blue lines indicate the 95% confidence intervals for the plot-lines. **Figure S11.** L0d3 branch after adding comparative published sequences. Tanzanian (dark green), Kuwait (Purple), other colours according to Figure 2. Yellow clade - southern African branch. Light green clade – Tanzanian and Kuwait branch. (DOC 3853 kb) Click here for file

Additional file 3: **Supplementary Methods.** (DOC 93 kb) Click here for file
