# Supplementary material for: MtDNA control region variation affirms diversity and deep sub-structure in populations from southern Africa
Source: BMC Evol Biol. 2013 Feb 27;13:56. doi: 10.1186/1471-2148-13-56 (PMC3607893; doi:10.1186/1471-2148-13-56)
Supplement: Additional file 1: Table S1. — Published mtDNA haplogroup and sub-haplogroup frequencies in San populations. Table S2. TMRCA calculated for the L0d/k subgroups. Four different mutation rates are applied. Table S3. Mismatch distribution statistics. Table S4. Mitochondrial population pairwise Fst values (All sequences). (DOC 149 kb) [file 1471-2148-13-56-S1.doc]

# Supplementary Tables

## Table S1: Published mtDNA haplogroup and sub-haplogroup frequencies in San populations

| **Main Haplogroup frequencies** | | | | | | |
| --- | --- | --- | --- | --- | --- | --- |
| **Haplogroup** | **Ju/’hoansi ^1^**  **(n=24)** | **!Xun ^2^**  **(n=43)** | | **Khwe ^2^**  **(n=31)** | **!Xun+Khwe ^3^**  **(n=18)** |  |
| L0d | 0.958 | 0.512 | | 0.161 | 0.611 |  |
| L0k | 0.042 | 0.256 | | 0.226 | 0.222 |  |
| L0a | - | 0.023 | | 0.097 | - |  |
| L1b | - | - | | 0.032 | - |  |
| L2* | - | - | | - | 0.167 |  |
| L2a | - | - | | 0.097 | - |  |
| L2b | - | 0.047 | | 0.065 | - |  |
| L3b | - | 0.116 | | 0.032 | - |  |
| L3e | - | 0.047 | | 0.290 | - |  |
| **Sub-Haplogroup frequencies as fractions of the total number of L0d/k haplotypes in the sample group (classified according to new nomenclature)** | | | | | | |
| **Sub-**  **Haplogroup** | **Ju/’hoansi ^1^**  **(n=24)** | **!Xun ^2^**  **(n=33)** | **Khwe ^2^**  **(n=12)** | | **!Xun+Khwe ^3^**  **(n=15** | **Bantu-speakers ^4,5^**  **(n=21)** |
| L0d1a | 0.083 | 0.091 | - | | 0.067 | - |
| L0d1b | 0.708 | 0.030 | - | | - | 0.190 |
| L0d1c | - | 0.485 | - | | 0.467 | 0.095 |
| L0d2a | 0.042 | - | - | | 0.067 | 0.143 |
| L0d2b | - | - | - | | - | 0.095 |
| L0d2c | 0.125 | - | - | | 0.067 | 0.238 |
| L0d2d | - | - | - | | - | 0.095 |
| L0d3 | - | - | - | | - | 0.048 |
| L0dx | - | 0.061 | 0.417 | | 0.067 | 0.048 |
| Unclassified | - | - | - | | - | 0.048 |
| L0k1 | 0.042 | 0.333 | 0.583 | | 0.267 | - |

^1^  Vigilant *et al.,* 1991

^2^  Chen *et al.,* 2000

^3^  Tishkoff *et al.,* 2007

^4^  Salas *et al.,* 2002

^5^  Perreira *et al.,* 2001

## Table S2: TMRCA calculated for the L0d/k subgroups. Four different mutation rates are applied

| **Split from other** | | | | | | | | | | | | | | | | |
| --- | --- | --- | --- | --- | --- | --- | --- | --- | --- | --- | --- | --- | --- | --- | --- | --- |
| **Ref** |  |  | **Horai ^1^** | | | **Soodyall ^2^** | | | **Ward ^3^** | | | | | **Foster ^4^** | | |
| **Haplogroup** | **ρ** | **σ** | **Years** | **SD** | | **Years** | **SD** | | | **Years** | | **SD** | | **Years** | **SD** | |
| L0d | 10.8580 | 2.2635 | 138002 | 28768 | | 116247 | 24233 | | | 96601 | | 20138 | | 53668 | 11188 | |
| L0d1a | 7.1351 | 2.0865 | 90685 | 26519 | | 76389 | 22338 | | | 63480 | | 18563 | | 35267 | 10313 | |
| L0d1b | 4.6709 | 1.5502 | 59366 | 19703 | | 50007 | 16597 | | | 41556 | | 13792 | | 23087 | 7662 | |
| L0d1c | 6.7119 | 2.2906 | 85306 | 29113 | | 71858 | 24523 | | | 59714 | | 20379 | | 33175 | 11322 | |
| L0d2a | 3.8242 | 1.7380 | 48604 | 22089 | | 40942 | 18607 | | | 34023 | | 15463 | | 18902 | 8590 | |
| L0d2b | 8.5000 | 2.4777 | 108032 | 31491 | | 91002 | 26527 | | | 75623 | | 22044 | | 42013 | 12247 | |
| L0d2c | 3.2941 | 1.5519 | 41867 | 19724 | | 35267 | 16615 | | | 29307 | | 13807 | | 16282 | 7671 | |
| L0d2d | 5.0000 | 1.8559 | 63549 | 23588 | | 53531 | 19869 | | | 44484 | | 16512 | | 24714 | 9173 | |
| L0d3 | 8.0000 | 2.6273 | 101678 | 33392 | | 85649 | 28128 | | | 71174 | | 23375 | | 39542 | 12986 | |
| L0dx | 4.0000 | 1.5411 | 50839 | 19587 | | 42824 | 16499 | | | 35587 | | 13711 | | 19771 | 7617 | |
| L0k1 | 8.5161 | 2.8144 | 108237 | 35770 | | 91174 | 30131 | | | 75766 | | 25039 | | 42093 | 13911 | |
|  |  |  |  | |  |  | |  | | |  | |  |  | |  |
| **TMRCA** | | | | | | | | | | | | | | | | |
|  | **ρ** | **σ** | **Years** | **SD** | | **Years** | **SD** | | | **Years** | | **SD** | | **Years** | **SD** | |
| L0d | 9.8580 | 2.0307 | 125292 | 25810 | | 105541 | 21741 | | | 87705 | | 18067 | | 48725 | 10037 | |
| L0d1 | 6.4286 | 1.2576 | 81706 | 15984 | | 68825 | 13464 | | | 57194 | | 11189 | | 31775 | 6216 | |
| L0d2 | 4.8718 | 1.6199 | 61919 | 20588 | | 52158 | 17343 | | | 43343 | | 14412 | | 24080 | 8007 | |
| L0d1a | 4.1351 | 1.1634 | 52556 | 14786 | | 44271 | 12455 | | | 36789 | | 10351 | | 20439 | 5750 | |
| L0d1b | 3.6709 | 1.1845 | 46656 | 15055 | | 39301 | 12681 | | | 32659 | | 10538 | | 18144 | 5855 | |
| L0d1c | 4.7119 | 1.8019 | 59887 | 22902 | | 50446 | 19291 | | | 41921 | | 16031 | | 23290 | 8906 | |
| L0d2a | 1.8242 | 1.0143 | 23185 | 12891 | | 19530 | 10859 | | | 16230 | | 9024 | | 9016 | 5013 | |
| L0d2b | 6.5000 | 2.0344 | 82613 | 25857 | | 69590 | 21780 | | | 57829 | | 18100 | | 32128 | 10055 | |
| L0d2c | 2.2941 | 1.1867 | 29157 | 15083 | | 24561 | 12705 | | | 20410 | | 10558 | | 11339 | 5866 | |
| L0d2d | 4.0000 | 1.5635 | 50839 | 19872 | | 42824 | 16739 | | | 35587 | | 13910 | | 19771 | 7728 | |
| L0d3 | 4.0000 | 1.7037 | 50839 | 21654 | | 42824 | 18240 | | | 35587 | | 15157 | | 19771 | 8421 | |
| L0dx | 3.0000 | 1.1726 | 38129 | 14903 | | 32118 | 12554 | | | 26690 | | 10432 | | 14828 | 5796 | |
| L0k1 | 1.5161 | 0.9596 | 19269 | 12197 | | 16232 | 10274 | | | 13488 | | 8538 | | 7494 | 4743 | |

Years are calculated from **ρ** by multiplying with the specific mutation rate

Standard deviation (SD) are calculated from **σ** by multiplying with the specific mutation rate

^1^  Horai *et al.,* (1995)

^2^  Soodyall *et al.*, (1996)

^3^  Ward *et al.,* (1991)

^4^  Foster *et al.,* (1996)

## Table S3: Mismatch distribution statistics

| **HG** | **Raggedness index** | **τ** | **T *** | **Model (SSD) p-value** |
| --- | --- | --- | --- | --- |
| L0d1a | 0.013 | 6.285 | 27 958 | 0.700 |
| L0d1b | 0.033 | 6.805 | 30 271 | 0.230 |
| L0d1c | 0.080 | 5.971 | 26 561 | 0.160 |
| L0d2a | 0.042 | 1.545 | 6 873 | 0.680 |
| L0d2b | 0.276 | - |  | 0.000# |
| L0d2c | 0.135 | - |  | 0.000# |
| L0d2d | 1.000 | - |  | 0.000+ |
| L0d3 | 0.053 | 4.500 | 20 018 | 0.600 |
| L0dx | 0.528 | - |  | 0.180# |
| L0k1 | 0.055 | 1.393 | 6 197 | 0.600 |
| L0a | 0.049 | 15.965 | 71 019 | 0.360 |
| M | 0.019 | 9.559 | 42 522 | 0.450 |
| R | 0.014 | 10.424 | 46 370 | 0.490 |

* T – Time before present that expansion took place (calculation explained in Supplementary Methods)

# expansion hypothesis rejected - 95% CI overlap

+ excluded – too few sequences

HG – Haplogroup, SSD - Sum of Squared deviation

## Table S4: Mitochondrial population pairwise Fst values (All sequences)

|  | AFR | CAC | COL | DRC | EUR | CNC | GUG | HER | IND | JOH | KAR | KHO | KWE | NAM | SOT | XUN | ZUX |
| --- | --- | --- | --- | --- | --- | --- | --- | --- | --- | --- | --- | --- | --- | --- | --- | --- | --- |
| AFR | 0.000 |  |  |  |  |  |  |  |  |  |  |  |  |  |  |  |  |
| CAC | 0.155 | 0.000 |  |  |  |  |  |  |  |  |  |  |  |  |  |  |  |
| COL | 0.240 | 0.010 | 0.000 |  |  |  |  |  |  |  |  |  |  |  |  |  |  |
| DRC | 0.078 | 0.110 | 0.193 | 0.000 |  |  |  |  |  |  |  |  |  |  |  |  |  |
| EUR | 0.031 | 0.273 | 0.326 | 0.206 | 0.000 |  |  |  |  |  |  |  |  |  |  |  |  |
| CNC | 0.398 | 0.094 | 0.031 | 0.365 | 0.495 | 0.000 |  |  |  |  |  |  |  |  |  |  |  |
| GUG | 0.457 | 0.204 | 0.149 | 0.418 | 0.563 | 0.128 | 0.000 |  |  |  |  |  |  |  |  |  |  |
| HER | 0.217 | 0.216 | 0.260 | 0.082 | 0.363 | 0.432 | 0.489 | 0.000 |  |  |  |  |  |  |  |  |  |
| IND | 0.034 | 0.213 | 0.278 | 0.110 | 0.082 | 0.433 | 0.489 | 0.245 | 0.000 |  |  |  |  |  |  |  |  |
| JOH | 0.393 | 0.129 | 0.089 | 0.346 | 0.495 | 0.078 | 0.108 | 0.410 | 0.435 | 0.000 |  |  |  |  |  |  |  |
| KAR | 0.518 | 0.187 | 0.081 | 0.487 | 0.633 | 0.036 | 0.263 | 0.557 | 0.547 | 0.179 | 0.000 |  |  |  |  |  |  |
| KHO | 0.468 | 0.152 | 0.073 | 0.450 | 0.567 | 0.016 | 0.182 | 0.511 | 0.505 | 0.122 | 0.057 | 0.000 |  |  |  |  |  |
| KWE | 0.211 | 0.055 | 0.106 | 0.092 | 0.320 | 0.227 | 0.272 | 0.193 | 0.258 | 0.165 | 0.319 | 0.308 | 0.000 |  |  |  |  |
| NAM | 0.322 | 0.033 | 0.012 | 0.257 | 0.436 | 0.023 | 0.147 | 0.307 | 0.366 | 0.057 | 0.084 | 0.057 | 0.128 | 0.000 |  |  |  |
| SOT | 0.127 | 0.006 | 0.063 | 0.044 | 0.229 | 0.193 | 0.268 | 0.129 | 0.177 | 0.208 | 0.277 | 0.274 | 0.024 | 0.113 | 0.000 |  |  |
| XUN | 0.428 | 0.178 | 0.125 | 0.369 | 0.523 | 0.125 | 0.079 | 0.428 | 0.461 | 0.038 | 0.204 | 0.180 | 0.172 | 0.102 | 0.235 | 0.000 |  |
| ZUX | 0.181 | 0.000 | 0.013 | 0.100 | 0.280 | 0.095 | 0.186 | 0.163 | 0.230 | 0.124 | 0.152 | 0.158 | 0.037 | 0.029 | 0.000 | 0.146 | 0.000 |
