# Supplementary material for: MtDNA control region variation affirms diversity and deep sub-structure in populations from southern Africa
Source: BMC Evol Biol. 2013 Feb 27;13:56. doi: 10.1186/1471-2148-13-56 (PMC3607893; doi:10.1186/1471-2148-13-56)

# Supplementary Figures

**Figure S1** Map indicating the place of origin for the Coloured, Khoe and San individuals who participated in the study. Labels and further information are available in Table 1. Bantu-speakers and people of non-African descent were sampled from various locations in South Africa and are not indicated on the map.


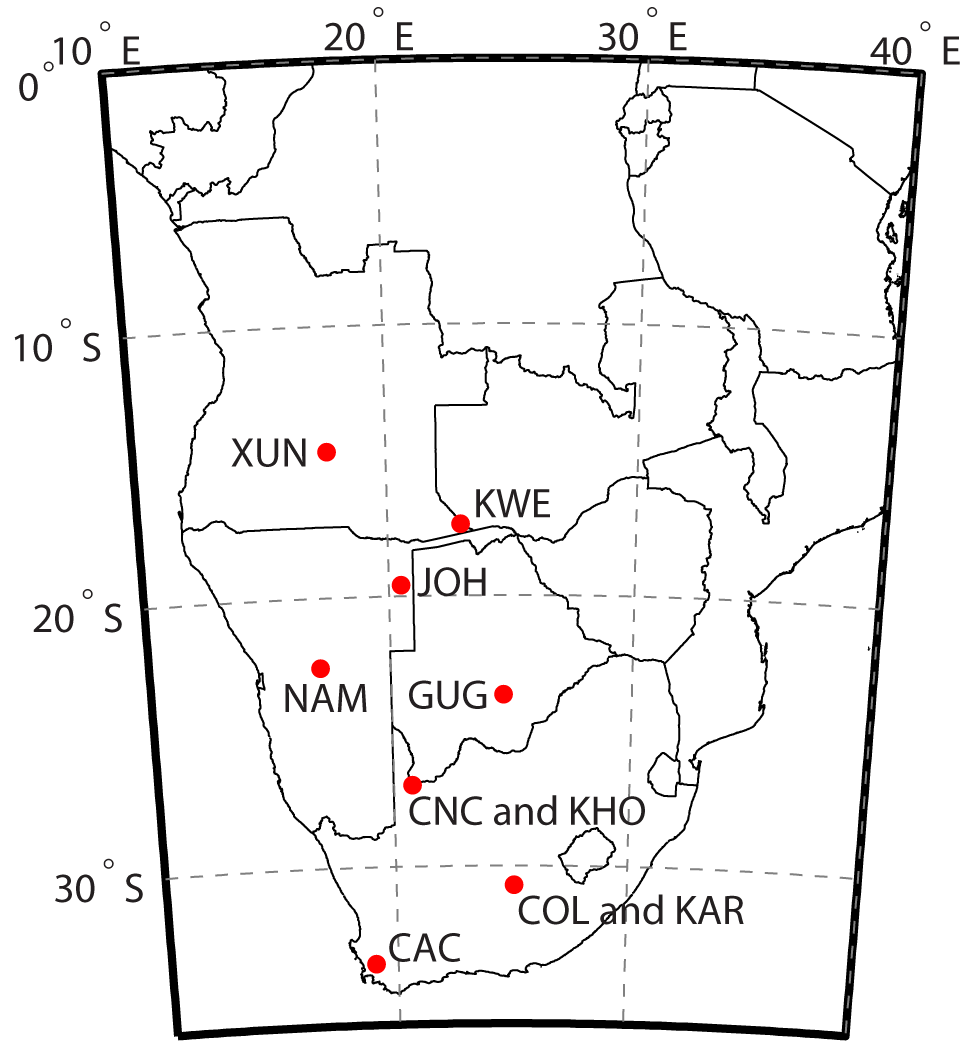


**Figure S2** Bar plot of percentage mtDNA haplogroup assignment in study populations


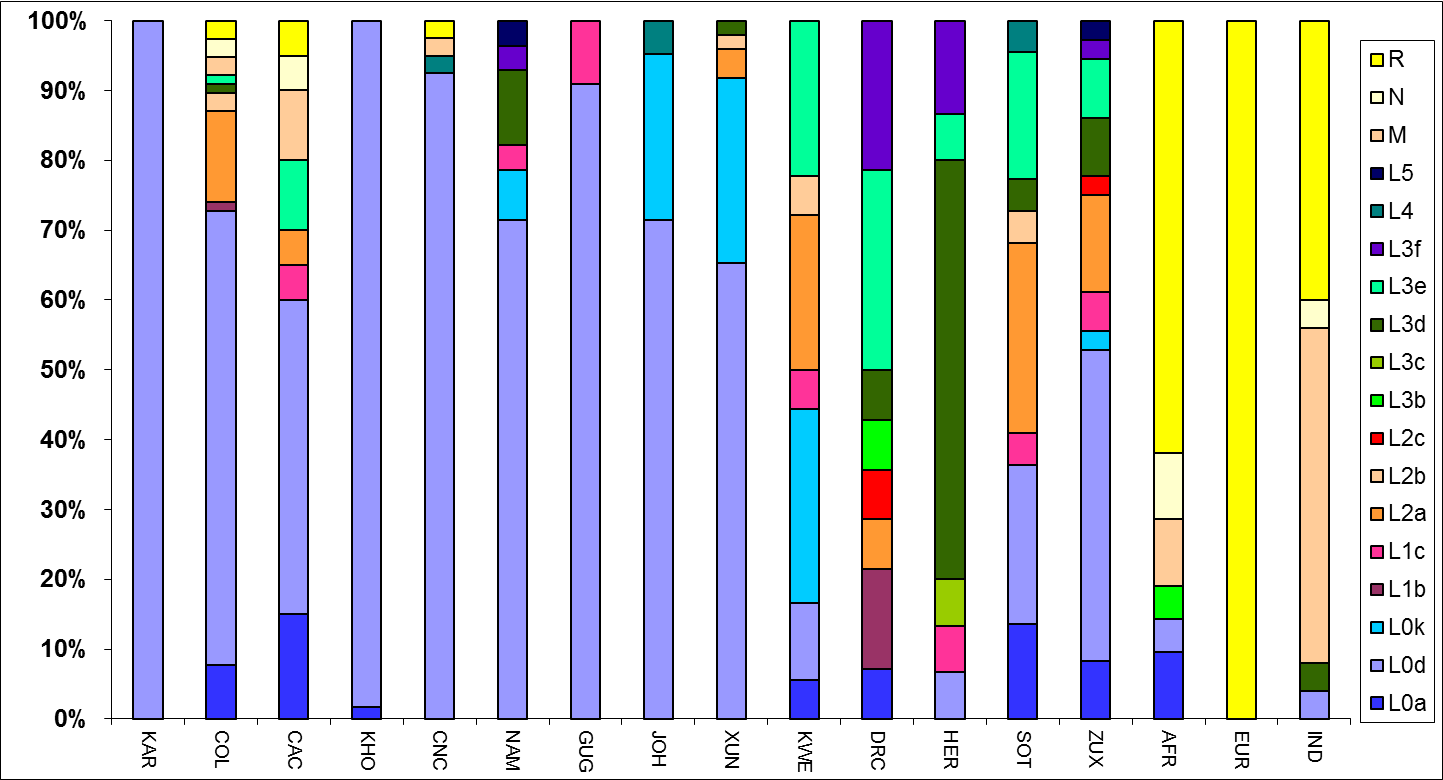

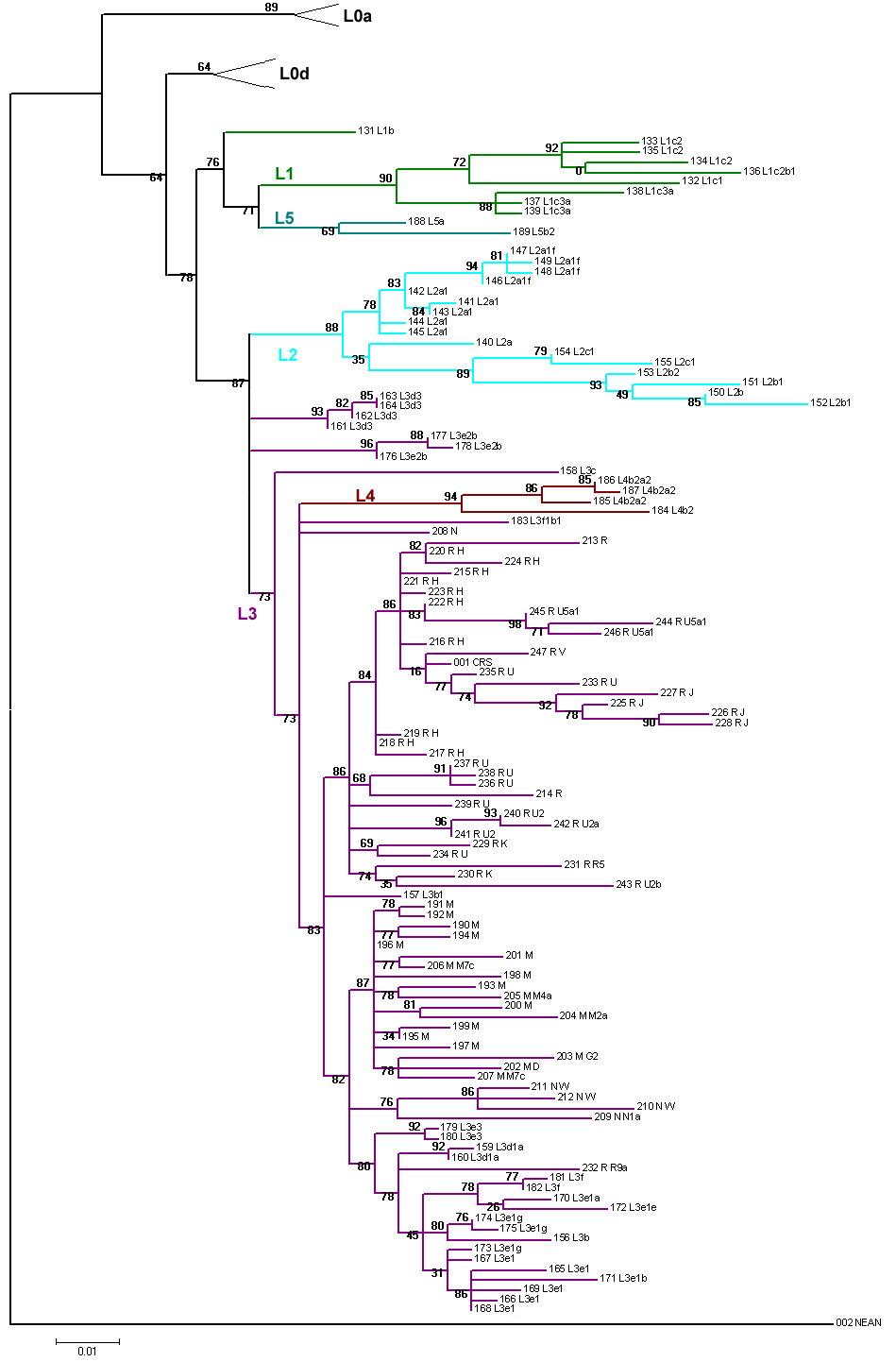


**Figure S3a** Maximum likelihood tree representing the substructure of L1 to L5. A Neanderthal sequence forms the outgroup. Branch support (%) was calculated through aLRT.

**Figure 3.5a** T


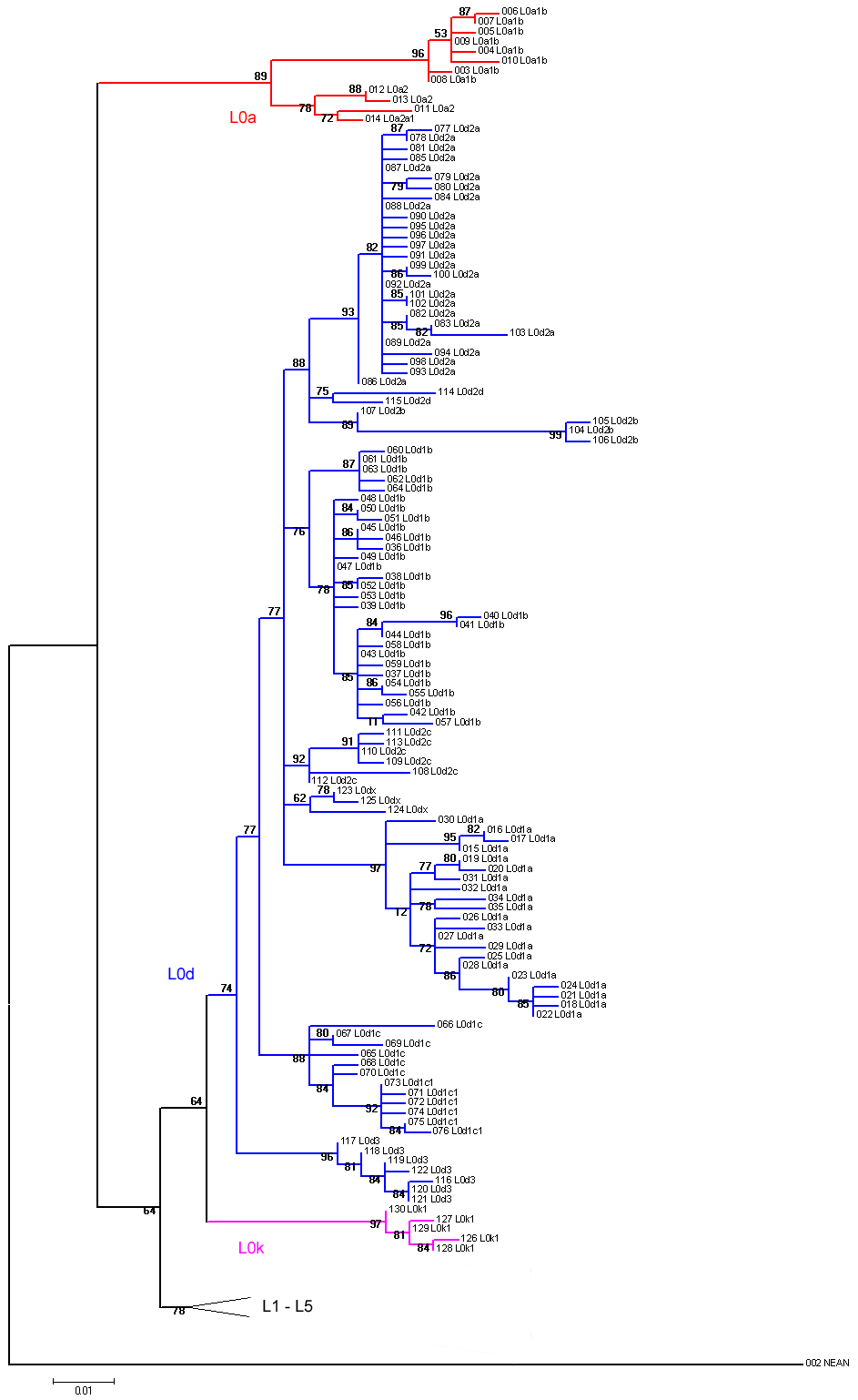


**Figure S3b** Maximum likelihood tree showing the relationships of the different mtDNA haplotypes within haplogroup L0. A Neanderthal sequence forms the outgroup. Branch support (%) was calculated through aLRT.

**Figure S4** Bar plot of percentage L0d/k sub-haplogroup assignment in study populations. Published comparative data are according to Table S1


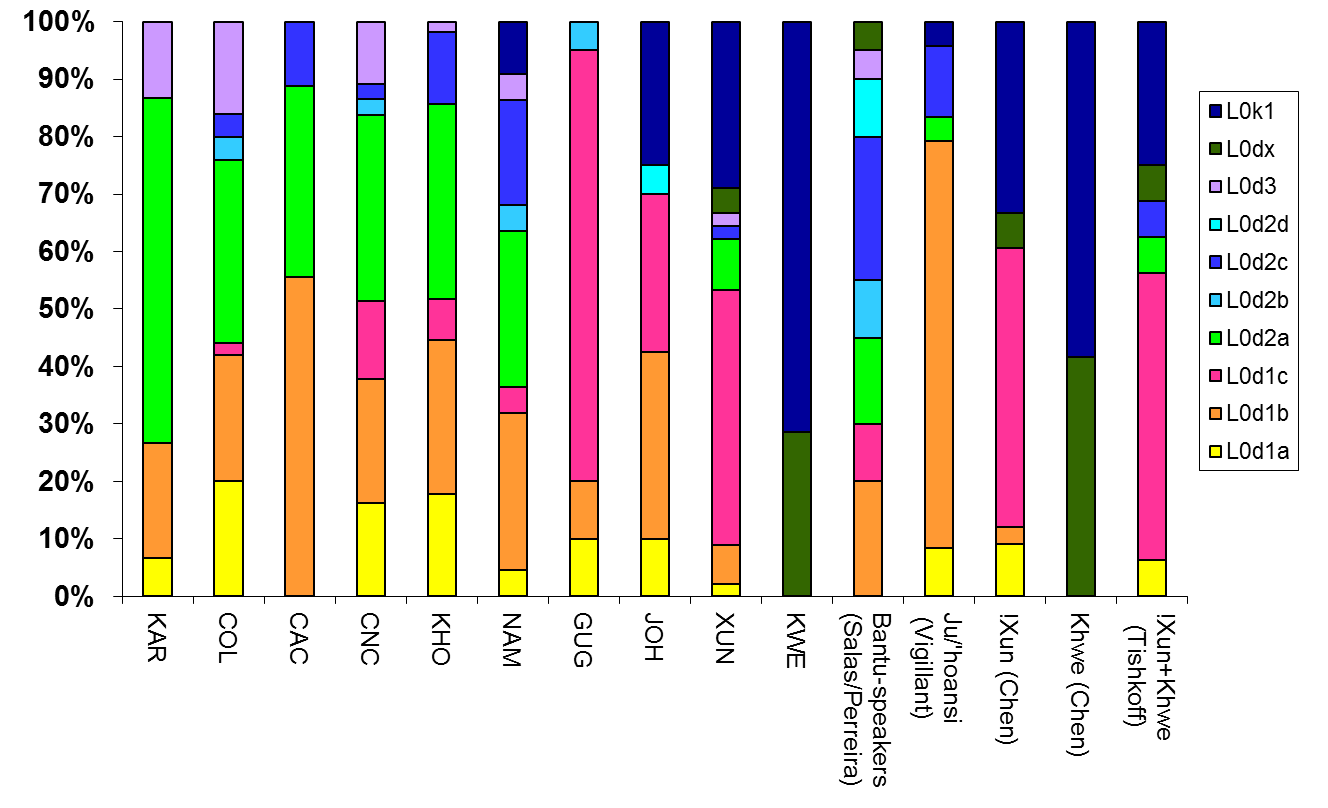


**Figure S5** Graphic representation of coalescent times and times of divergence of the mtDNA sub-haplogroups of L0d and L0k (according to Table 2). The mutation rate estimated by Ward *et al.,* (1991) was used in these estimates.


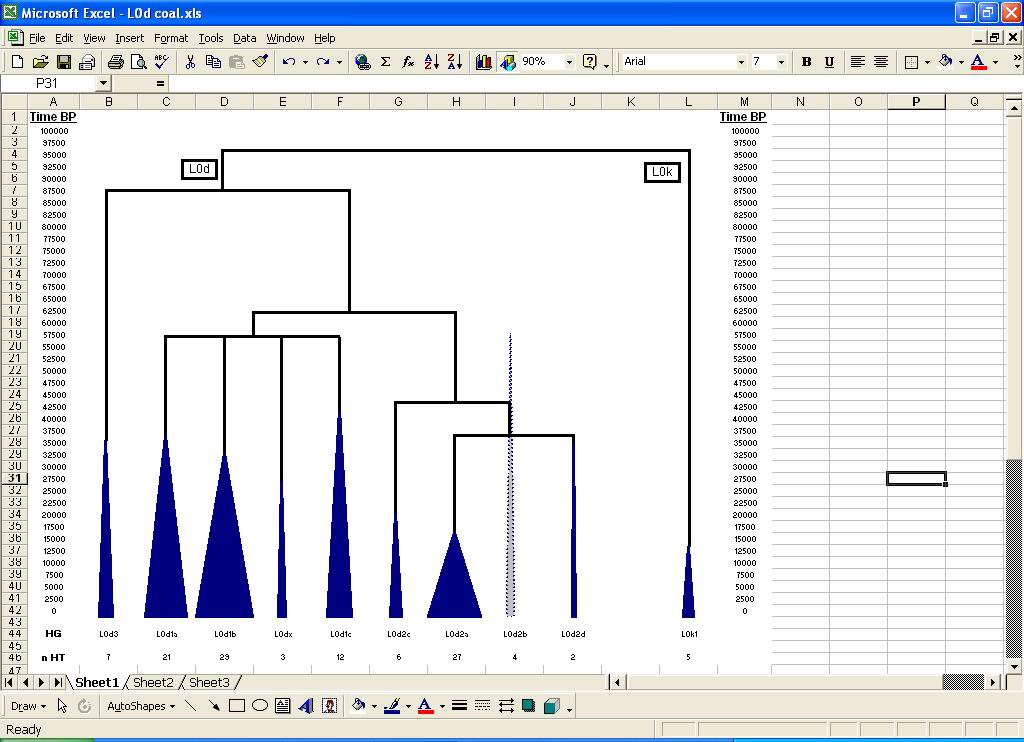

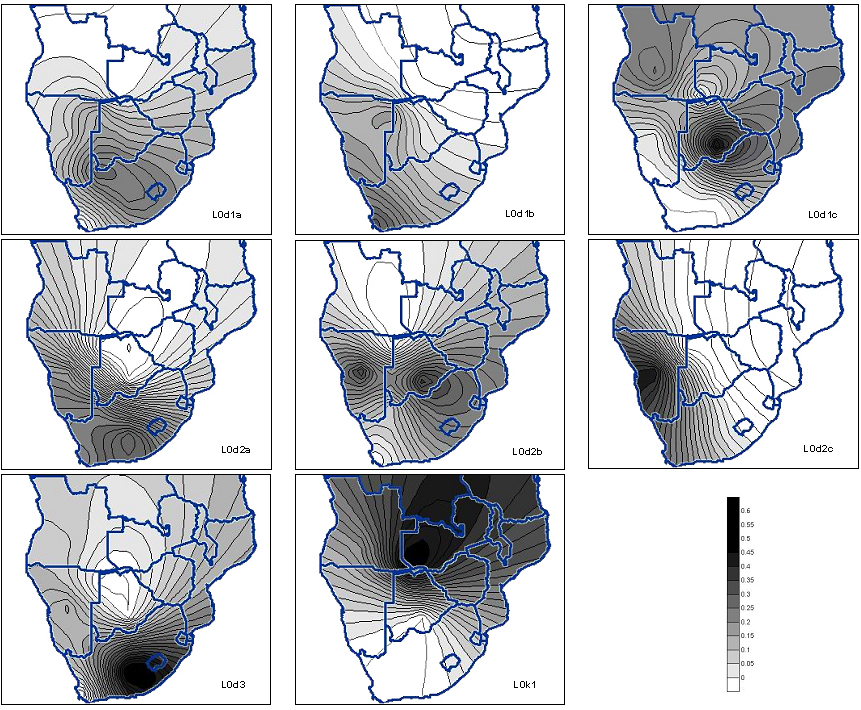


**Figure S6** Contour plots indicating the frequency distributions of L0d/k subgroups


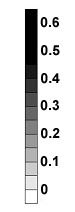


**Figure S7** Contour plots of L0d1c split into two subgroups, L0d1c1 and the remaining L0d1c sequences (L0d1c-).


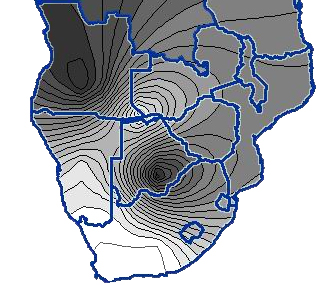


**L0d1c1**


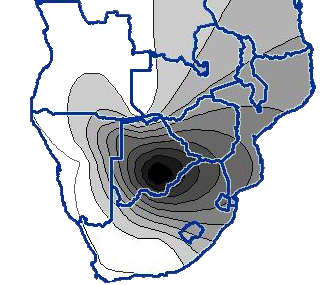


**L0d1c-**


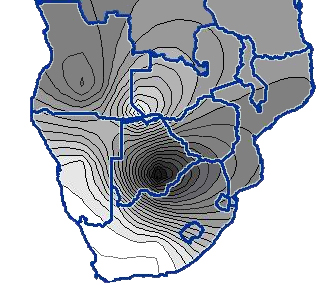


**L0d1c**

**A**

**B**

**C**

**Figure S8** Mismatch distributions of L0d/k sub-haplogroups and comparative groups. # expansion hypothesis rejected (95% CI overlap)


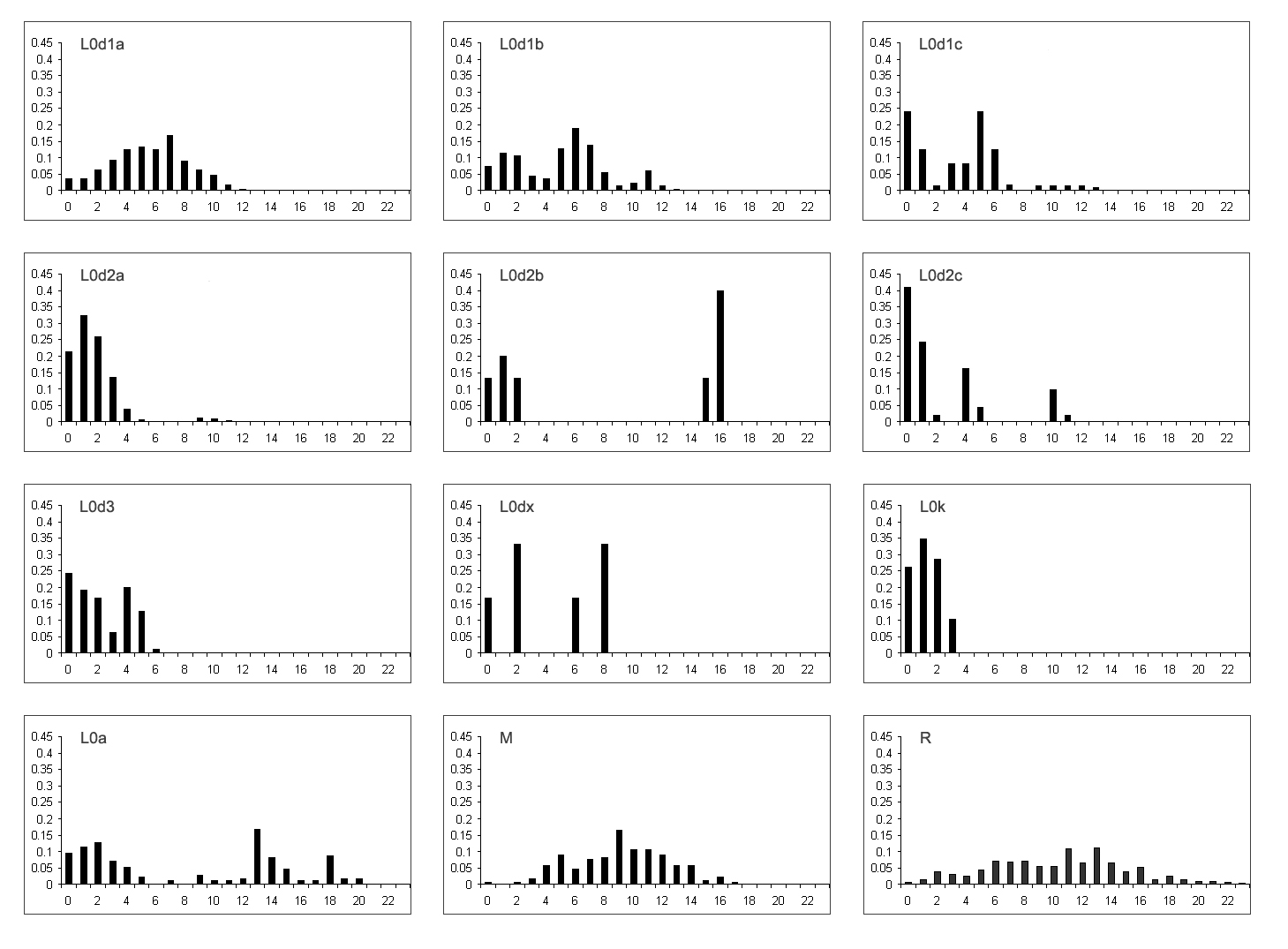


#

#

#

**Figure S9** Bayesian Skyline plots of L0d haplogroups showing changes in *N_e_* through time. *N_e_* is represented on the Y-axis, while years before present are represented on the X-axis, with the present indicated by 0. L0d3+ is L0d3 including the east African and Kuwait sequences. L0d3- includes only L0d3 sequences from the present study. The black bold vertical lines indicate the coalescence date and the lighter vertical lines the 95% confidence intervals for the coalescence. The blue lines indicate the 95% confidence intervals for the plot-lines


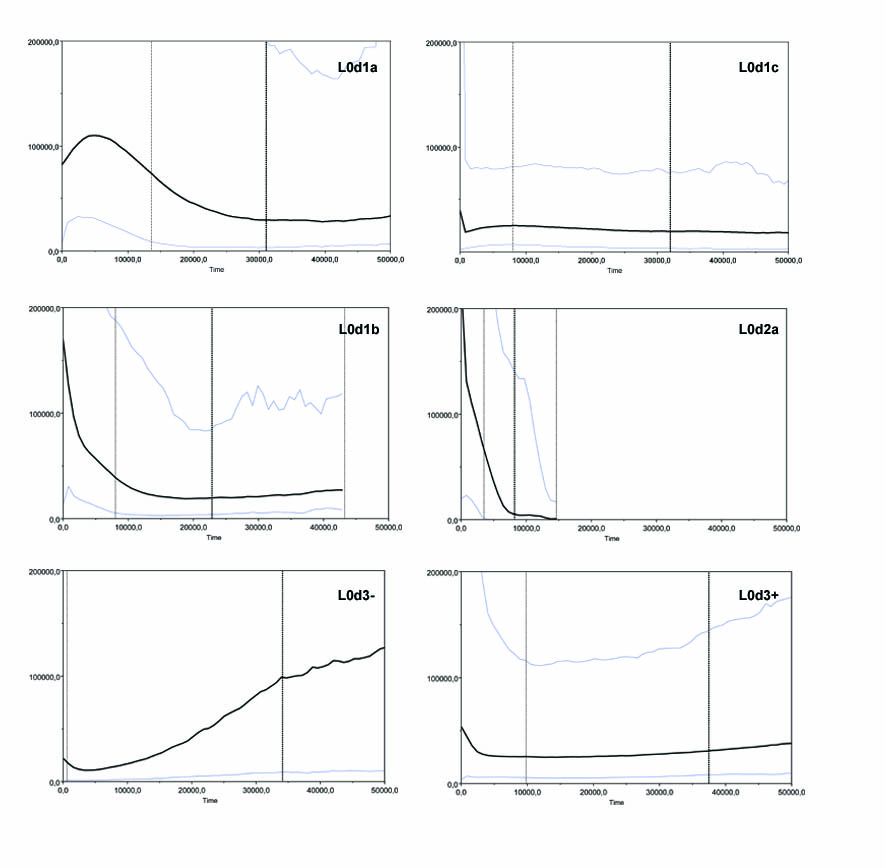


**Figure S10** Bayesian Skyline plots of all L0d haplotypes across all population groups, showing changes in *N_e_* through time. *N_e_* is represented on the Y-axis, while years before present are represented on the X-axis, with the present indicated by 0. The black bold vertical lines indicate the coalescence date and the lighter vertical lines the 95% confidence intervals for the coalescence. The blue lines indicate the 95% confidence intervals for the plot-lines.


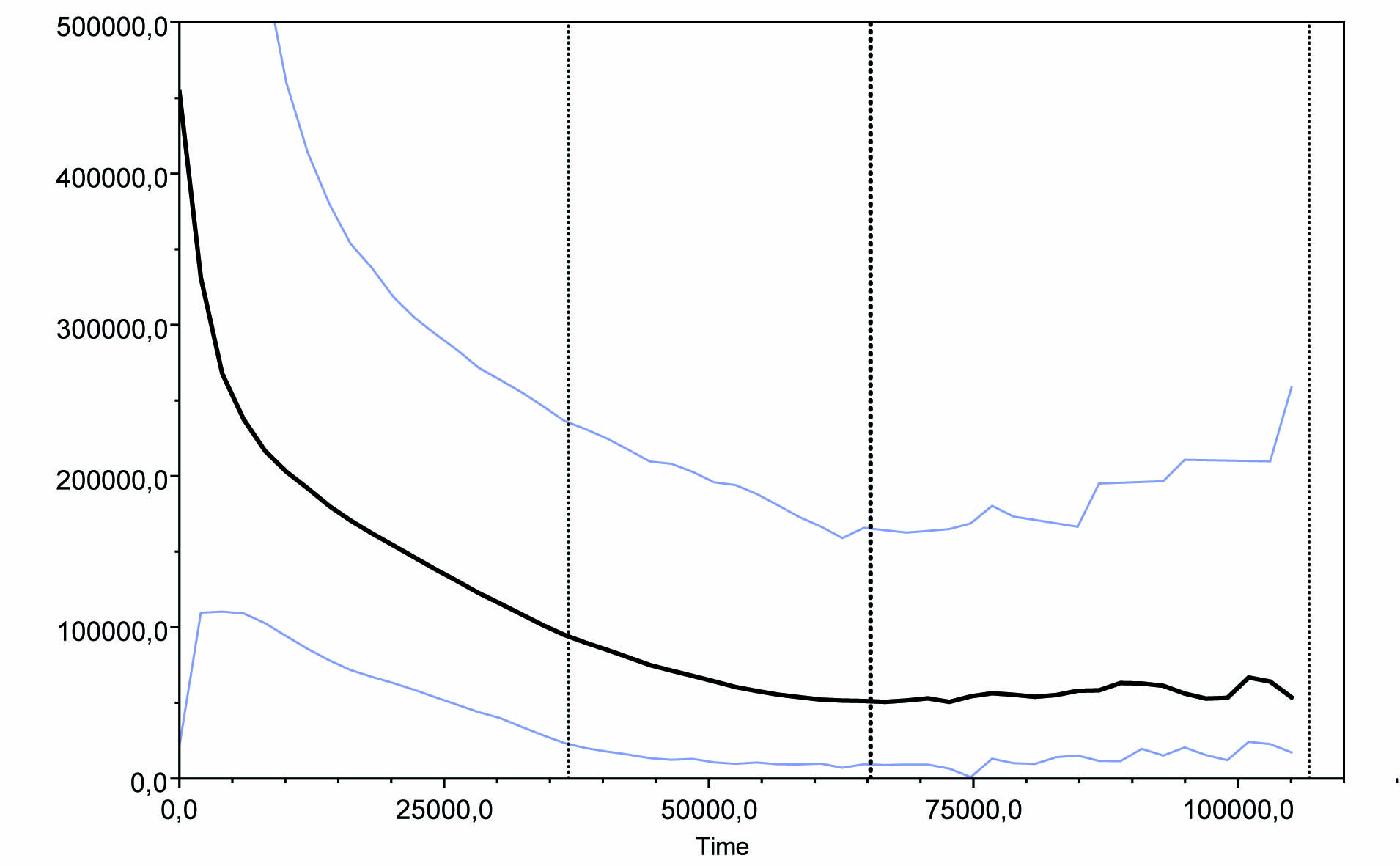


**Figure S11** L0d3 branch after adding comparative published sequences. Tanzanian (dark green), Kuwait (Purple), other colours according to Figure 2. Yellow clade - southern African branch. Light green clade – Tanzanian and Kuwait branch


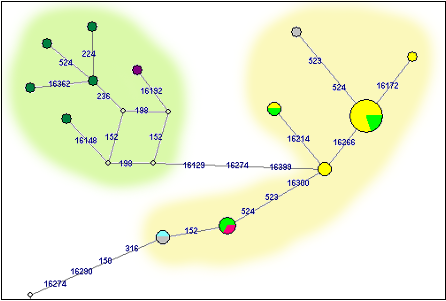

Supplement: Additional file 2: Figure S1. — Map indicating the place of origin for the Coloured, Khoe and San individuals who participated in the study. Labels and further information are available in Table 1. Bantu-speakers and people of non-African descent were sampled from various locations in South Africa and are not indicated on the map. Figure S2. Bar plot of percentage mtDNA haplogroup assignment in study populations. Figure S3a. Maximum likelihood tree representing the substructure of L1 to L5. A Neanderthal sequence forms the outgroup. Branch support (%) was calculated through aLRT. Figure S3b. Maximum likelihood tree showing the relationships of the different mtDNA haplotypes within haplogroup L0. A Neanderthal sequence forms the outgroup. Branch support (%) was calculated through aLRT. Figure S4. Bar plot of percentage L0d/k sub-haplogroup assignment in study populations. Published comparative data is according to Table S1. Figure S5. Graphic representation of coalescent times and times of divergence of the mtDNA sub-haplogroups of L0d and L0k (according to Table 2). The mutation rate estimated by Ward et al., (1991) was used in these estimates. Figure S6. Contour plots indicating the frequency distributions of L0d/k subgroups. Figure S7. Contour plots of L0d1c split into two subgroups, L0d1c1 and the remaining L0d1c sequences (L0d1c-). Figure S8. Mismatch distributions of L0d/k sub-haplogroups and comparative groups. # expansion hypothesis rejected (95% CI overlap). Figure S9. Bayesian Skyline plots of L0d haplogroups showing changes in Ne through time. Ne is represented on the Y-axis, while years before present are represented on the X-axis, with the present indicated by 0. L0d3+ is L0d3 including the east African and Kuwait sequences. L0d3- includes only L0d3 sequences from the present study. The black bold vertical lines indicate the coalescence date and the lighter vertical lines the 95% confidence intervals for the coalescence. The blue lines indicate the 95% confidence intervals for the plot [file 1471-2148-13-56-S2.doc]
