# Supplementary material for: MtDNA control region variation affirms diversity and deep sub-structure in populations from southern Africa
Source: BMC Evol Biol. 2013 Feb 27;13:56. doi: 10.1186/1471-2148-13-56 (PMC3607893; doi:10.1186/1471-2148-13-56)
Supplement: Additional file 3: — Supplementary Methods. (DOC 93 kb) [file 1471-2148-13-56-S3.doc]

# Supplementary Methods (Analyses and parameters)

## Maximum Likelihood tree

A Maximum likelihood tree for unique haplotypes was constructed using PHYML [1]. The HKY substitution model, with Gamma distributed rates and Invariable sites, received the best likelihood prediction through likelihood ratio tests using MODELTEST v.3.7 [2] in conjunction with PAUP v.4.0b10 [3] and were implemented in the Maximum likelihood analysis. The tree topology search employed was nearest neighbour interchange (NNI). An approximate likelihood ratio test (aLRT) was computed to determine branch support [4]. Trees were visualized in MEGA4 [5].

## Median Joining Networks

Networks of the sequences were constructed using the Median Joining algorithm [6] of NETWORK v.4.5.0.0 [7]. Networks were subjected to maximum parsimony post-analysis using the Steiner maximum parsimony algorithm [8] within NETWORK v.4.5.0.0. For network analysis the epsilon parameter was set to 2 and transversions were weighted 3x the weight of transitions. Furthermore the weight of the 16189 position was reduced 10x and the weight each of the CA repeats at position 523 was reduced 5x per nucleotide in the repeat.

## TMRCA calculations

Time to most recent common ancestor (TMRCA) of the L0d haplogroup and the L0d sub-haplogroups was calculated from the median joining network using the Rho statistic [9]. A mutation rate of 2.5 x 10^-6^ per nucleotide per generation [10] was assumed. This mutation rate is also similar to the widely used rate of Soares *et al.,* [11]; which is given as 9.883 × 10^-8^ mutations per nucleotide per year for whole of the control region and therefore amounts to 2.47 x 10^-6^ per nucleotide per generation (compared to 2.5 x 10^-6^ per nucleotide per generation of Ward *et al.,* [10]). Time estimates were also calculated using other published mutation rates (i.e. 1.75 x 10^-6^ per nucleotide per generation [12]; 4.5 x 10^-6^ per nucleotide per generation [9]; 2.1 x 10^-6^ per nucleotide per generation [13] but because of its intermediate value the mutation rate of Ward *et al.,* [10] (which is similar to the Soares *et al.,* [11] mutation rate) was used in subsequent discussions and analyses. A generation time of 25 years was used throughout.

## Mismatch distributions

Mismatch distributions of populations and haplogroups were calculated in ARLEQUIN v.3.11 [14]. From these the validity of demographic expansions and the date of expansions were inferred. The demographic expansion scenario is tested through simulating a population going through an expansion and testing whether the actual data is significantly different from the simulated expansion scenario. A non-significant Sum of Squared deviation (SSD) p-value will therefore indicate a population/group of sequences that went through an expansion. Parameters calculated are θ_1_ , θ_0_ , and τ. τ gives an indication of the time of the expansion. The mutation rate of 2.5 x 10^-6^ per nucleotide per generation [10] and a generation time of 25 years were used to convert τ (Tau) to T (Time BP when expansion took place) by using the equation T= (τ/2μ) x generation time. In the equation μ is the mutation rate per gene per generation (2.5 x 10^-6^ per nucleotide per generation [10] x 1124 sites results in μ = 2.81 x 10^-3^).

## Haplogroup isofrequency maps

Haplogroup isofrequency maps were generated applying the Kriging method [15, 16] incorporated in the SURFER v.8.06.39 [17]. Mitochondrial contour plots were based on frequencies of the L0d/k subgroups on the background of the L0d/k group as a whole. This was done to eliminate the effects that admixture from Bantu-speakers and non-Africans would have on the distribution of the L0d/k subgroups. When frequencies were calculated, sample size effects were corrected by adjusting the total sample sizes in all groups to the same value.

## Bayesian Skyline Plots

To visually represent the effective population size changes through time, Bayesian Skyline Plots (BSP) [18] were constructed. For each of the haplogroups, BSPs of effective population size through time were constructed using a Markov Chain Monte Carlo (MCMC) sampling algorithm, as implemented in BEAST v.1.4.8 [19]. The population size function of the BSP can be implemented using either a piecewise constant or a piecewise linear function of population size change. In the present study, a piecewise linear model made up of 10 control points was used. The general time-reversible (GTR) substitution model with estimated base frequencies and a Gamma + Invariant Sites heterogeneity model was used to infer the ancestral gene trees for each haplogroup. The mean substitution rate was fixed to the rate of Ward et al., [10] and a relaxed molecular clock (Uncorrelated Lognormal) was employed. Each MCMC sampling was repeated for 40 000 000 generations, sampled every 4 000, with the first 4 000 000 generations discarded as burn-in. All runs had an effective sample size of at least 1 000 for the parameters of interest. Each independent run was repeated at least twice and results were combined using the LOGCOMBINER v.1.4.8 tool included in the BEAST package. BSPs were visualized in TRACER v.1.4 [20].

## Summary statistics and neutrality tests

The summary statistics; number of sequences, haplotype number, gene diversity [21] and nucleotide diversity [21], for haplogroups were calculated in DNASP v.4.10 [22]. The population mutation parameter (θ) was estimated from using segregating sites (θs per nucleotide site) as well as the Waterson estimator (W-θs per sequence) [23]. The selective neutrality tests of Tajima’s D [24], Fu’s Fs statistic [25] and the R2 statistic [26] were also calculated using DNASP v.4.10.

## Population pairwise Fst

Population pairwise differences were calculated with ARLEQUIN v3.11 [14] by using Fst [27] incorporating the nucleotide correction model of Tamura and Nei [28] and a gamma correction of 0.532. Groups with N<10 were excluded from the population analyses (Table 1). The distance matrices were visualized through UPGMA trees in PAST v.1.54 [29].

## Genetic vs. geographic distance comparison

The relationships between geographic and genetic distances for different population groups were investigated by doing a linear regression in R. The regression was applied to a scatter plot resulting from pairwise comparisons of distance matrices based on geographic and genetic distances (Fst). The linear regression model involved fitting a straight line with a gradient to the graph and recording significance values to the model and model variables (such as the gradient). Additionally, a Mantel test implemented in ARLEQUIN v.3.11 [14] was also done to test the correlation between the geographic and genetic distance matrices.

The geographic distance matrix was constructed by obtaining latitude and longitude information of the different sampling locations from the website “Google Maps Latitude, Longitude Popup” [30] and calculating the great circle distance (in km) between the points using the “Latitude/Longitude Distance Calculation” [31].

# Supplementary Methods References
